# Hydrogeological characterization of an alpine aquifer system in the Canadian Rocky Mountains

**DOI:** 10.1007/s10040-020-02153-7

**Published:** 2020-05-04

**Authors:** Craig William Christensen, Masaki Hayashi, Laurence R. Bentley

**Affiliations:** 1 Department of Geoscience, University of Calgary, Calgary, Alberta Canada; 2 Norwegian Geotechnical Institute, Oslo, Norway

**Keywords:** Geophysical methods, Geomorphology, Talus, Moraine, Canada

## Abstract

Groundwater storage in alpine regions is essential for maintaining baseflows in mountain streams. Recent studies have shown that common alpine landforms (e.g., talus and moraine) have substantial groundwater storage capacity, but the hydrogeological connectivity between individual landforms has not been understood. This study characterizes the hydrogeology of an alpine cirque basin in the Canadian Rocky Mountains that contains typical alpine landforms (talus, meadow, moraines) and hydrological features (tarn, streams, and springs). Geological, hydrological, and hydrochemical observations were used to understand the overall hydrogeological setting of the study basin, and three different geophysical methods (electrical resistivity tomography, seismic refraction tomography, and ground penetrating radar) were used to characterize the subsurface structure and connectivity, and to develop a hydrogeological conceptual model. Geophysical imaging shows that the talus is typically 20–40 m thick and highly heterogeneous. The meadow sediments are only up to 11 m thick but are part of a 30–40-m-thick accumulation of unconsolidated material that fills a bedrock overdeepening (i.e. a closed, subglacial basin). A minor, shallow groundwater system feeds springs on the talus and streams on the meadow, whereas a deep system in the moraine supplies most of the water to the basin outlet springs, thereby serving as a ‘gate keeper’ of the basin. Although the hydrologic functions of the talus in this study are substantially different from other locations, primarily due to differences in bedrock lithology and geomorphic processes, the general conceptual framework developed in this study is expected to be applicable to other alpine regions.

**Electronic supplementary material:**

The online version of this article (10.1007/s10040-020-02153-7) contains supplementary material, which is available to authorized users.

## Résumé

Dans les régions alpines, le stockage des eaux souterraines est essentiel au maintien du débit de base des cours d’eau de montagne. De récentes études ont montré que les formes de relief alpin courants (par exemple le talus et la moraine) ont une capacité de stockage des eaux souterraines substantielle, mais la connectivité hydrogéologique entre les formes particulières de relief n’a pas été comprise. La présente étude caractérise l’hydrogéologie d’un bassin d’un cirque alpin des Montagnes Rocheuses Canadiennes qui présente des formes typiques de relief (talus, prairie, moraines) et des caractéristiques hydrogéologiques (petits lacs, cours d’eau et sources). Des observations géologiques, hydrologiques et hydrochimiques ont été utilisées pour comprendre le contexte hydrogéologique général du bassin étudié, et trois méthodes géophysiques différentes (tomographie par résistivité électrique, tomographie par sismique réfraction et géoradar) ont été utilisées pour caractériser la structure et la connectivité du sous-sol et pour développer un modèle conceptuel hydrogéologique. L’imagerie géophysique montre que le talus a généralement une épaisseur de 20–40 m et est fortement hétérogène. Les sédiments de la prairie n’ont que 11 m d’épaisseur mais font partie d’une accumulation épaisse de 30–40 m de matériel non consolidé, qui remplit un surcreusement profond dans le substratum (c-à-d, un bassin fermé sous-glaciaire). Un petit système aquifère phréatique nourrit des sources sur le talus et des cours d’eau sur la prairie, tandis que le système profond de la moraine fournit la plus grande partie de l’eau aux sources sortant du bassin, servant ainsi de « portier » du bassin. Bien que les fonctions hydrologiques du talus soient dans cette étude substantiellement différentes de celles d’autres localisations, surtout en raison des différences de lithologie du socle et des processus géomorphologiques, on escompte que le schéma conceptuel général développé ici est applicable à d’autres régions alpines.

## Resumen

El almacenamiento de aguas subterráneas en las regiones alpinas es esencial para mantener los flujos de base en los cursos de agua de las montañas. Estudios recientes han demostrado que las formas de relieve alpino comunes (por ejemplo, el talud y la morena) tienen una capacidad importante de almacenamiento de aguas subterráneas, pero no se ha comprendido la interrelación hidrogeológica entre las distintas formas de relieve. Este estudio caracteriza la hidrogeología de una cuenca de circo alpino en las Rocky Mountains del Canadá que contiene las típicas formas de relieve alpino (talud, pradera, morenas) y características hidrológicas (lagunas, arroyos y manantiales). Se utilizaron observaciones geológicas, hidrológicas e hidroquímicas para comprender el contexto hidrogeológico general de la cuenca de estudio, y se emplearon tres métodos geofísicos diferentes (tomografía de resistividad eléctrica, tomografía de refracción sísmica y georradar) para caracterizar la estructura y la conectividad en el subsuelo, y para elaborar un modelo conceptual hidrogeológico. Las imágenes geofísicas muestran que el talud suele tener un espesor de 20 a 40 m y es muy heterogéneo. Los sedimentos de la pradera sólo tienen un espesor de hasta 11 m, pero forman parte de una acumulación de material no consolidado de 30–40 m de espesor que rellena un basamento sobreexcavado (es decir, una cuenca subglacial cerrada). Un sistema menor de aguas subterráneas poco profundas alimenta los manantiales en el talud y los arroyos en la pradera, mientras que un sistema profundo en la morena suministra la mayor parte del agua a los manantiales de salida de la cuenca, sirviendo, así como “portadora” de la cuenca. Aunque las funciones hidrológicas del talud en este estudio son sustancialmente diferentes de las de otros lugares, principalmente debido a las diferencias en la litología del basamento y los procesos geomórficos, se espera que el marco conceptual general desarrollado en este estudio sea aplicable a otras regiones alpinas.

## 摘要

高山区地下水储量对于维持山区河流的基流至关重要。最近的研究表明,普通的高山地貌(例如碎石堆和冰碛石)具有重要的地下水储存能力,但尚未认识单个地貌间的水文地质的连通性。本研究描述了加拿大落基山脉高山圆形山谷盆地的水文地质特征,该盆地包含典型的高山地貌(碎石堆,草甸,冰碛石)和水文特征(冰斗湖,溪流和泉水)。利用地质,水文和水化学观测来了解研究区的整体水文地质环境,并使用三种不同的地球物理方法(电阻率层析成像,地震折射层析成像和探地雷达)来表征地下结构和连通性,以及建立水文地质概念模型。地球物理成像显示碎石层的厚度通常为20–40 m,并且高度不均匀。草甸沉积物的厚度仅为11 m,但属于30–40 m厚的未固结堆积物的一部分,这些未固结物质充满了深层的基岩(即封闭的亚冰川盆地)。少量的浅层地下水系统补充给碎石层上的泉水和草甸层的溪流,而冰碛土中的深层地下水系统则向盆地的出露泉水补给了大量水,从而成为盆地的“守门人”。尽管本研究中碎石层的水文功能与其他位置有很大不同,这主要是由于基岩岩性和地貌过程的差异所致,但本研究中开发的总体概念框架有望应用于其他高山地区。

## Resumo

O armazenamento de águas subterrâneas em regiões alpinas é essencial para manter os fluxos de base nos córregos das montanhas. Estudos recentes mostraram que formas de relevo alpinas comuns (por exemplo, tálus e morena) têm capacidade substancial de armazenamento de águas subterrâneas, mas a conectividade hidrogeológica entre formas de relevo individuais não foi compreendida. Este estudo caracteriza a hidrogeologia de uma bacia de circo alpino nas Montanhas Rochosas Canadenses que contém formas de relevo alpinas típicas (talus, prados, morenas) e características hidrológicas (tarn, córregos e nascentes). Observações geológicas, hidrológicas e hidroquímicas foram usadas para entender o cenário hidrogeológico geral da bacia em estudo e três métodos geofísicos diferentes (tomografia de resistividade elétrica, tomografia de refração sísmica e radar de penetração no solo) foram usados ​​para caracterizar a estrutura e a conectividade do subsolo, e desenvolver um modelo conceitual hidrogeológico. As imagens geofísicas mostram que o tálus tem tipicamente 20–40 m de espessura e é altamente heterogêneo. Os sedimentos dos prados têm apenas 11 m de espessura, mas fazem parte de um acúmulo de material não consolidado com 30–40 m de espessura que preenche um afundamento no leito de rocha (isto é, uma bacia subglacial fechada). Um sistema menor de águas subterrâneas rasas alimenta fontes no tálus e córregos no prado, enquanto um sistema profundo na morena fornece a maior parte da água às fontes de saída da bacia, servindo assim como um ‘guardião’ da bacia. Embora as funções hidrológicas do tálus neste estudo sejam substancialmente diferentes de outros locais, principalmente devido a diferenças na litologia dos leitos de rocha e nos processos geomórficos, espera-se que o quadro conceitual geral desenvolvido neste estudo seja aplicável a outras regiões alpinas.

## Introduction

Mountains are popularly referred to as the “water towers of the world” (Bandyopadhyay et al. 1997). Despite covering only approximately 25% of the world’s land surface area, they account for somewhere between 32% (Meybeck et al. 2001) and 60% (Bandyopadhyay et al. 1997) of surface runoff. Moreover, 7% of the world’s mountain regions provide essential water resources to downstream populations, and another 37% provide an important supporting supply in regions prone to shortages (Viviroli et al. 2007). Earlier spring freshets and decreased fall and winter flows have been documented over the course of the last half-century (e.g., Burn et al. 2008; Rood et al. 2008; DeBeer et al. 2016). This is concerning for both the water resources for and for mountain ecosystems, which exhibit both high biodiversity and high sensitivity to climatic changes (Hannah et al. 2007; Brown et al. 2009; Finn et al. 2013; Steinbauer et al. 2016)

Predicting streamflow changes in mountain regions is complicated by small-scale heterogeneity of alpine zones (DeBeer et al. 2016). In recent years, researchers have become increasingly aware of the important role groundwater plays in mountain hydrology. Whereas earlier workers assumed that mountain catchments behaved like “Teflon basins” (Clow et al. 2003), research since the mid-1990’s has demonstrated that even thin, coarse sediments in alpine zones constitute an important flow path and reservoir (e.g., Campbell et al. 1995; Mast et al. 1995).

Talus deposits, which are common, long-lasting alpine landforms, are important hydrogeological units in alpine zones. Several tracer studies from the Colorado Front Ranges of the Rocky Mountains showed that talus slopes are the primary storage reservoir in some basins (Davinroy 2000; Clow et al. 2003). Similar patterns have been observed in the Cordillera Blanca in Peru, where talus slopes (and related debris fans) are important recharge features, storing meltwater for the dry season (Baraer et al. 2014; Gordon et al. 2015). While some authors have found saturated thicknesses as great as 11 m (Clow et al. 2003), most concluded that groundwater flow in talus is concentrated in a thin layer along the bedrock–talus interface (Caballero et al. 2002; Muir et al. 2011; Volze 2015). For this reason, bedrock topography is thought to be an important control of flow paths and storage in talus and similar coarse-grained units in alpine terrain (Langston et al. 2011; McClymont et al. 2012; Harrington et al. 2018). Other authors have shown that the presence of interstitial ice and the proportion of fine-grained sediments can also have an effect (Davinroy 2000; Caballero et al. 2002).

Alpine meadows and wetlands have also attracted considerable attention. Though they do not store as much water as other types of landforms (e.g., Baraer et al. 2014), they serve other important ecological and hydrological functions. These units generally have more stable water tables, providing important habitat and buffering release of water to streams (Orellana et al. 2012). Normally, these areas discharge groundwater to streams, or are at least groundwater through-flow areas (Lord et al. 2011; Jin et al. 2012; Payn et al. 2012; Somers et al. 2016). Only rarely are they groundwater sinks; for example, only 4% of the wetlands inventoried by Fryjoff-Hung and Viers (2013) in the western United States were sinks. Recent work has shed light on what factors control the hydrogeology of these landforms, including bedrock topography, degree of stream incision, and hydraulic properties of the meadow sediments (Loheide and Gorelick 2007; McClymont et al. 2010; Essaid and Hill 2014; Gordon et al. 2015; Ciruzzi and Lowry 2017).

This article presents a hydrogeological conceptual model of an alpine headwater basin consisting of several landform units (talus, moraine, and meadow) based on hydrogeological and geophysical observations. The results add to the diversity of known groundwater regimes in alpine catchments and contribute to improve the broad understanding of alpine hydrology. The paper highlights how the hydrology of this site contrasts with other sites and presents an explanation of how differing geology and geomorphic processes led to the differences in hydrology. These insights will be helpful for building hydrogeological conceptual models in other alpine basins, and eventually improving our capability to identify the streams that are buffered against the variability in meteorological conditions.

## Study site

### Overview and bedrock geology

**Fig. 1:**
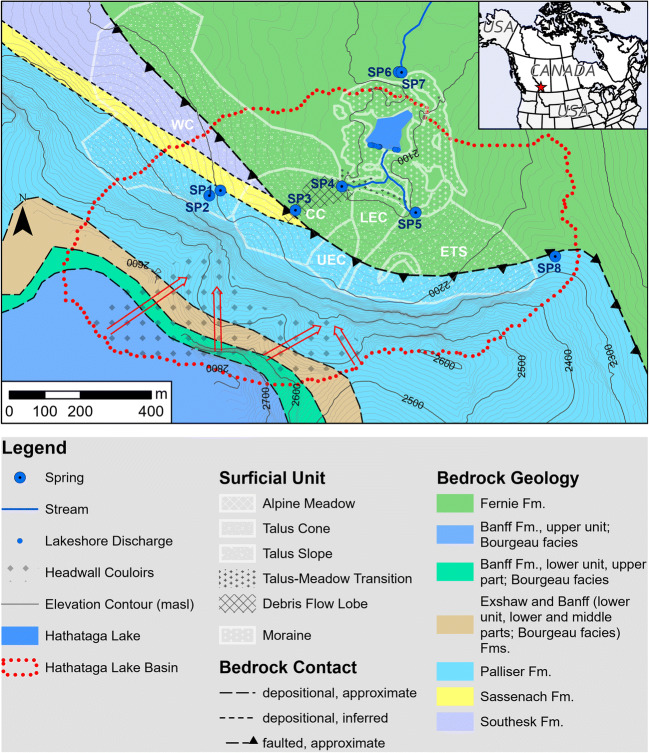
Map of Hathataga Lake Basin near Fortress Mountain showing bedrock lithology, surficial geology, and hydrology. White lettering notes the abbreviated names of the West Cone (WC), Central Cone (CC), Upper East Cone (UEC), Lower East Cone (LEC), and Eastern Talus Slope (ETS), and the red arrows denote the drainage direction within the headwall couloirs. Red star in the inset map shows the location of the site within Canada. Bedrock spatial data from McMechan (2012)

**Fig. 2:**
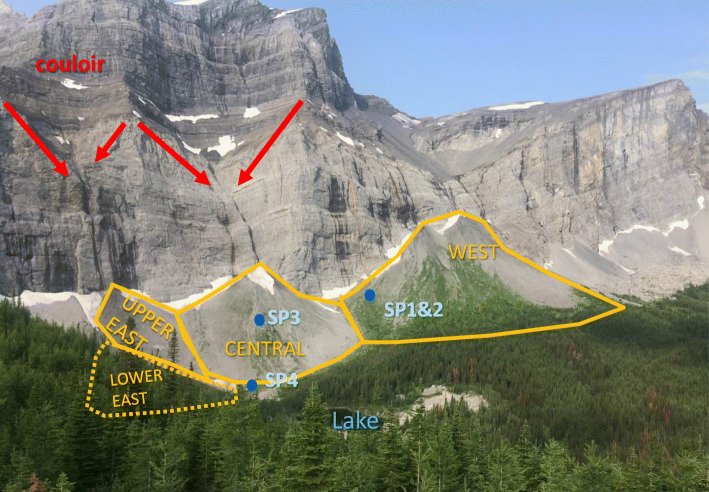
An oblique photo (facing southwest) of the talus deposits in the study area taken on July 26, 2018. Orange lines outline the four talus cones. (The Lower East cone is obscured by trees). Also noted are important springs (blue circles) and couloirs above the Upper East and Central Cones (red arrows)

The study was conducted in a north-facing cirque in the Front Ranges of the Canadian Rocky Mountains, situated in a former ski area called Fortress (50°49′14″ N, 115°12′50″ W; Fig. 1). It is located in the headwater region of the South Saskatchewan River, which receives on average 38% of annual flow from mountain zones (Ashmore and Church 2001; Viviroli and Weingartner 2004). The roads built for the ski area operation offer a relatively easy access to the high-elevation terrain, providing a unique opportunity for hydrogeological research. This study focuses on the complex terrain consisting of talus slopes, a meadow, and moraines encircling a tarn. The tarn does not have an official name, but it is located in a valley called Hathataga in the language of the local Stoney people (Crawler et al. 1987). Therefore, for the purpose of this paper, the tarn is called Hathataga Lake. This cirque is apparently drained by a perennial spring complex (SP6 and SP7 in Fig. 1) with no surface connection to the lake. A stream originating from the spring complex drains into Galatea Creek, which in turn drains into the Kananaskis River, and then the Bow River. The basin drained by the spring complex, which covers 0.92 km^2^, is herein referred to as the Hathataga Lake basin. Elevations in the basin range from 2,082 m above sea level (masl) at the outlet spring to 2,900 masl at the southern summits forming the headwall (Figs. 1 and 2). Annual total precipitation recorded at a weather station located 40 m northwest of SP6 was 780 mm in 2016, 1,180 mm in 2017, and 1,010 mm in 2018, and mean monthly temperature was −8.6 °C in January and 11.0 °C in July (J. Pomeroy, University of Saskatchewan, unpublished data, 2017).

Most of the Hathataga Lake basin is underlain by the highly recessive Jurassic-age Fernie Formation comprised primarily of highly fissile shale, but with some sandstone strata (Stockmal 1979; McMechan 2012; Fig. 1). The Palliser Formation, a Devonian-age carbonate rock, is the thickest unit within the headwall cliff (Stockmal 1979). Above this (2,500–2,900 masl) are the Upper Devonian- to Carboniferous-age Exshaw Formation and Carboniferous-age Banff Formation. The formations include dark black and incompetent shales, calcareous shale, and argillaceous lime wackestone (Stockmal 1979).

### Talus deposits

Four identifiable talus cones occur along the southern headwall (Fig. 2) and are referred to herein as the Upper East Cone (UEC), Lower East Cone (LEC), Central Cone (CC), and West Cone (WC). Both the UEC and CC occur below a narrow couloir, whereas the WC is below a much broader incised valley. The Eastern Talus Slope (ETS) is found at the southeast margin of the basin and has no obvious convex transverse profile nor a couloir concentrating rockfall. Grain sizes vary considerably throughout the talus, ranging from “coarse, openwork matrix with large void spaces” to “clast-supported with all voids filled” (Selby 1982). Completely matrix-supported deposits were not observed on the talus cones, but transitional zones where boulders are mixed in with fine sediments do occur between meadow and talus deposits (Fig. 1). Grain size distributions not only vary between cones, but also within them—for example, in the CC, fine-grained material is more common along the path of steepest descent from the apex of the cone to SP4 (labelled as a “debris flow lobe” in Fig. 1). In contrast, the western half of the CC is very coarse, blocky, and loosely packed. Photographs of various types of talus sediments can be found in Fig. S1 of the electronic supplementary material (ESM).

White’s (1981) classification scheme (Table S1 of the ESM) was used to determine which of the three main sediment redistribution processes were responsible for the formation of the talus—snow avalanche, rockfall, or alluvial processes. All cones have slopes steeper than 30° at their apex indicating rockfall and alluvial processes but are also concave upward at the base indicating alluvial processes and avalanche. Fine-grained sediments are washed in between coarser material at locations proximal to the apex of cones, indicating alluvial processes, yet a fringe of coarser debris is present at the toe of most indicating rockfall and avalanche. Some areas lack vegetation and have angular fragments indicating recent rockfall, yet others are vegetated and have clear fluvial features like channels or debris flow lobes located below couloirs that concentrate water flow. Moreover, in winter, large cornices develop atop the headwall causing avalanches; hence, all three of the main redistribution processes contributed to the formation of these deposits, with the relative importance of each differing depending on the location within the talus cones. Several springs occur at various positions within talus cones and slopes (Figs. 1 and 2). Their hydrogeological characteristics are described in the results section.

### Meadow and Hathataga Lake

The grassy meadow at the base of the talus slopes covers approximately 5,000 m^2^. This mostly flat expanse has an elevation range between 2,094 and 2,096 masl, sloping gently up with a concave curvature as it transitions gradually into the adjacent talus deposits (Fig. 1). Moraines border the north side of meadow and surround Hathataga Lake (Fig. 1). Most of the moraines surrounding Hathataga Lake have been interpreted as recessional moraines given their poorly sorted angular grains (Knight 2003). While the moraines may be covered by vegetation in some places, the soil cover in these areas is no more than 2 m thick. There is also a small moraine on the ETS.

Hathataga Lake is a seasonal water body, which forms during spring melt and dries out almost completely by late summer. When lake levels are low, groundwater discharge is visible along the south shore through several discrete points at an approximate elevation of 2,090 masl (Fig. 1). The water flowing from these discharge points has a relatively stable temperature ranging between 2 and 5 °C throughout the summer. Though most of the lakebed is covered by a layer of fine-grained sediment, its thickness is not uniform. In most areas, this bed, which separates the lake water from the coarse morainic material below, is only a few centimetres or decimetres thick. When water levels are low, water can be seen discharging to the coarse-grained sediment below via gaps in this fine-sediment layer present at the north end of the lake (Fig. S2 of the ESM).

## Methods

### Hydrogeological measurements, water sampling, and analysis

**Fig. 3:**
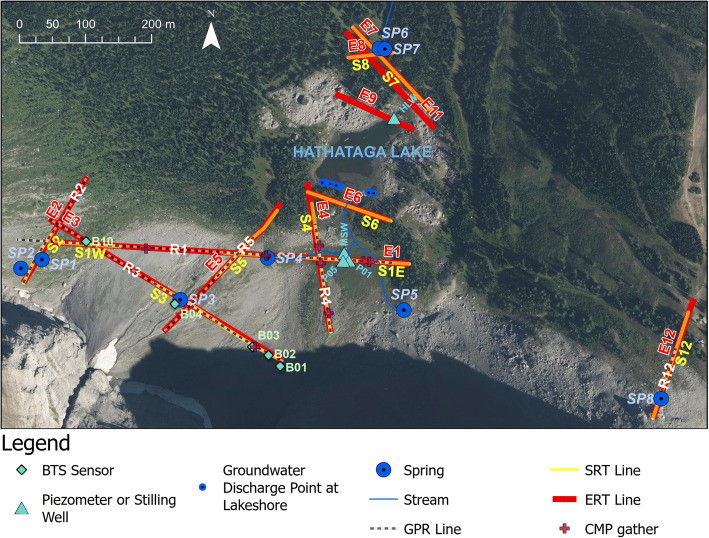
Map showing the locations of geophysical lines and other sensors. Note that ERT lines are labeled “EX”, SRT lines labeled “SX”, and GPR lines labeled “RX”, with “X” being the line number. Line S1 has two segments: S1W at the west and S1E to the east. Air photos from Alberta Environment and Park (2013)

Discharge in the meadow stream was measured periodically in July 2015 just before it entered Hathataga Lake (Fig. 3) using a horizontal-axis current meter (Global Water, FP101) and the velocity-area method (Dingman 2002, pp. 609). Piezometers were installed at two locations (P01 and P05 in Fig. 3) to document seasonal changes in the water table and examine the interaction between the meadow stream and groundwater. These were constructed from 2.5-cm-diameter PVC pipes with the bottom 20–30 cm perforated and wrapped with a fine wire mesh. They were installed in a 5-cm-diameter holes drilled using a hand-auger, and the annulus space was filled with coarse sand over the perforated interval and bentonite clay from the top of the screen up to surface. Self-logging pressure transducers (Solinst, Levelogger 3001) were used to monitor water level at 15-min intervals during July–September 2016 in the piezometers and two stilling wells: one in the meadow stream (MSW in Fig. 3) and another in the north end of Hathataga Lake (HLW).

Water samples were collected during July 14–28, 2015 from the springs, the meadow stream, Hathataga Lake, and groundwater discharge points on the south shore of the lake using a 50-ml syringe. The samples were filtered through a 0.45-um-diameter membrane filter and stored in high-density polyethylene bottles kept in a refrigerator. The samples were analyzed for alkalinity by titration, major-ion concentrations by ion-exchange chromatography, and ^2^H/^1^H and ^18^O/^16^O isotopic ratios by a cavity ring-down spectroscopy isotope analyzer and reported as δ-values.

### Geophysical imaging: electrical resistivity tomography

Geophysical imaging was conducted using electrical resistivity tomography (ERT), seismic refraction tomography (SRT), and ground-penetrating radar (GPR) along survey lines shown in Fig. 3. Most data were acquired during July 13–31, 2015, except for E11 acquired in a follow-up campaign in July 2016.

Resistivity data were collected using the IRIS Instruments Syscal Pro data acquisition system and a modified dipole-dipole array, which had an *n*-spacing of up to 6 (Fig. S3 of the ESM) and included partial reciprocals (roughly 40%). Most lines used 4-m electrode spacing, but spacings ranged between 1 and 8 m (see Table S2 in the ESM for key survey statistics). Electrode locations were measured using a differential global positioning system unit (Leica Geosystems AG, Leica Viva CS15) with an Alberta Survey Control Marker (No. 486340) as a base station. Location uncertainty was generally <3 cm, though errors were higher in densely forested areas due to poor signal reception. Such instances were usually isolated, so spurious values were removed, and a more realistic position was interpolated from surrounding points.

A consistent challenge with resistivity data acquisition was poor electrode contact, especially in portions of the talus lacking fine-grained sediments. Contact resistance was reduced below 20 kΩ in most cases by using saltwater-soaked sponges or bentonite clay to improve the contact between electrodes and rocks. Some electrodes that proved difficult to remedy were accepted with a contact resistance of 20–50 kΩ, and those above 50 kΩ were excluded from the survey.

The raw data were subject to a consistent quality control protocol. All negative resistances and were removed. The instrument has a precision of 0.1 mV, so potential measurements of 0.1 mV or less were also discarded. Measurements that exceeded a certain variance threshold during the transmitter’s on-time were rejected. A threshold of 5% standard deviation for line 2 and 1% in all other cases was used. Finally, any remaining, isolated outliers (as viewed in pseudo-depth versus resistivity semi-log plots) were removed manually.

The quality-controlled data were inverted using RES2DINV (Geotomo Software 2012). The use of the square of the data misfit (the L2 norm) tends to make the inversion scheme sensitive to bad data points (Farquharson and Oldenburg 1998) which is an issue in areas on the talus or moraine with poor electrode contact; hence, the absolute values of data misfit (the L1 norm) was employed (see the ESM for exact mathematical formulation).

A model grid cell width equal to half the unit electrode spacing was used to reduce the influence of high-resistivity anomalies in the thin, dry, uppermost layers. Applying slightly more horizontal than vertical smoothing (i.e. a vertical-to-horizontal smoothing ratio of 0.7) tended to produce models with lower data misfit and were deemed more geologically plausible given the depositional environment. The mean absolute percent error was used to assess the goodness of fit of the resulting models and ranged between 3.3 and 15%.

### Geophysical imaging: seismic refraction tomography

Seismic data were collected using the Geometrics Geode data acquisition system with a geophone spacing of 2 m in most cases except S7 (2.5 m) and S8 (1 m). Spike geophones were used where there was sufficient fine-grained sediment to insert the spikes, and plate geophones were used on talus and moraine deposits where there were no fine-grained sediments. Rocks were stacked on the geophones to minimize noise from wind-induced movement and to help improve coupling. A 5-kg sledgehammer striking a 22-kg cylindrical aluminum plate was used as the seismic energy source, which was placed every six geophones (12 m). Hammer blows were repeated (i.e. stacked) seven to thirteen times until there was no noticeable change in the stacked signal. Total line length varied from 71 to 286 m (see Table S3 of the ESM for key survey parameters).

First arrival times were manually picked in ReflexW software (Sandmeier Software 2005). Given the low energy of the source, first breaks were often difficult to pick at far-offset geophones, especially in loose talus material. To assist with picking first breaks in these cases, band-pass filtering was used, whereby the highest-frequency noise was removed first with a relatively wide band-pass filter, and then with a second, narrower one (Christensen et al. 2017). P-wave velocity models were produced using the program of Lanz et al. (1998), which uses a fast finite-difference eikonal solver in its forward model (see the ESM for exact mathematical formulation). Two grids of differing resolutions are used in these series of computations. A coarser one is used for defining the slowness model, while a finer one is used in the forward modelling. Zhi (2013) tested a wide range of grid setups using this same inversion scheme and found that the optimal model grid has cells between 75 to 100% of the nominal geophone spacing; smaller values led to unstable inversion results. The optimal computational grid used 16 cells (a 4 × 4 grid) per model parameter cell.

P-wave velocity tomograms were produced using a consistent workflow. First, a simple straight-line analysis (e.g. Lowrie 1997, pp. 145–147) was used to estimate the depth to major velocity boundaries and guide selection of an appropriate starting model for inversion. With this software, using initial models that favour shallower ray paths usually leads to more accurate models because they help detect shallow layers and ensures that ray paths enter model cells from many different angles (Musil et al. 2002). In the first run of the inversion, a high dampening ratio (relative to the starting model) and a model with a linearly increasing velocity with depth was used. The result of this first run was then used as the new starting model in the second and final run, and the dampening ratio reduced. The root mean squared (RMS) error was used as the mean summary statistic to assess the goodness of fit between the data and output models and ranged from 1.5 to 4.6 ms.

### Geophysical imaging: ground penetrating radar

Ground-penetrating radar data were acquired using the Sensors & Software PulseEkko Pro system with low frequency (50 MHz) antennae to achieve a sufficient depth of penetration and to avoid excessive diffractions from large boulders in talus slopes. Data were only collected in unforested areas to avoid reflected signals from trees, using a 0.25-m step size with a 2-m antenna separation. Signals were digitized at a 0.8-ns sampling interval, and 32 stacks were used per capture. In addition, nine common midpoint (CMP) surveys were performed to acquire velocity information. Each began with a 2-m antenna separation and had a step size of 0.2 m (i.e. 0.1-m movement away from the midpoint for each antenna) between captures.

Data were processed in ReflexW (Sandmeier Software 2005) using a standard workflow: Dewow filtering, static correction, and an exponential gain function. Noisy traces, likely caused by interference from intermittent use of short-range radios by the survey crew, were manually removed. The direct air and ground arrivals remain in the processed images, meaning the upper 3 m of radargrams in the talus and 1 m of radargrams in more electrically conductive sediments are artefacts and not real structures.

The CMP velocity analysis module of ReflexW was then used to develop one-dimensional (1D) velocity models from the CMP surveys. In this module, a velocity semblance analysis is first carried out, producing a two-dimensional (2D) plot of semblance values as function of depth and root mean square (RMS) velocities. Next, the user manually picks RMS velocities that best recreate the reflectors seen in the original CMP data. From these picks of RMS velocity, the software calculates a 1D velocity model. The final output velocities ranged between 0.025 m ns^−1^ in the middle of the meadow to 0.13 m ns^−1^ in the driest, most open-work portions of the talus.

A correlation between resistivity and electromagnetic (EM) wave velocity was noted (Fig. S4 in the ESM), which is to be expected since there are large variations in near-surface water content on site, and water content is a strong control on both geophysical properties. The correlation was used to develop a 2D EM velocity model based on ERT images and which was used to convert GPR reflection sections from time to depth sections. However, this velocity model is still imperfect, with measured values typically ranging ±0.025 m ns^−1^ around the modeled correlation in Fig. S4 in the ESM. Both the semblance analysis results and ERT surveys have limits with resolution, and other geological parameters beyond water content such as soil and rock type, can affect resistivity and EM-wave velocity differently. For these reasons, uncertainty exists in the EM velocity which is estimated to be ±0.025 m ns^–1.^ In depth-converted radargrams, the positional uncertainty is proportional with to depth, meaning that the vertical position uncertainty of deeper portions of the images are larger than near-surface portions. Since the velocity model uncertainty remains a roughly constant ±0.025 m ns^−1^, materials with low EM-wave velocities tend to have a greater depth uncertainty, being as high as 50% in the wet, fine-grained portions of the meadow.

In the study of alpine landforms like talus, moraine, and rock glaciers, migration is not typically used in the processing of GPR images (Sass 2006; Sass et al. 2010; McClymont et al. 2010, 2011, 2012; Langston et al. 2011; Muir et al. 2011). McClymont et al. (2011) found that migration led to a degradation of image quality and a loss of internal structures and attributed this to out-of-plane effects. Similarly, Lukas and Sass (2010) noted that it is the highly heterogeneous nature of sediments in these landforms that make migration with a single, constant velocity untenable. Unmigrated GPR images are therefore used for interpreting subsurface structures in this study, with image texture being the focus of interpretations. The precise location and geometry of major reflectors is only used where there is sufficient supporting evidence from other geophysical methods to confirm their existence.

### Bottom temperature of snowpack measurement

Bottom temperature of snowpack (BTS) is an indicator of the likelihood of permafrost occurrence, when it is measured in mid- to late-winter under a sufficiently thick (> 1 m) snow cover that insulates the effect of air temperature. Hoelzle et al. (1999) found that BTS < −3 °C corresponds to zones where permafrost was probable, while BTS > −2 °C corresponds to zones where it was improbable. Self-logging temperature sensors (Maxim, iButton DS1921Z) were installed at five locations (Fig. 3) on October 22, 2015 and recorded the ground surface temperature at 3-h intervals until June 14, 2016. The “zero-curtain”, the period during which spring melt where temperatures remain a constant 0 °C, was used to calibrate sensors (Outcalt et al. 1990; Staub et al. 2015). The average temperature during February–March 2016 was used as BTS as snow depth in the area reached >1 m by February 1 at the weather station near SP6 (J. Pomeroy, University of Saskatchewan, unpublished data, 2016).

## Results

### Hydrogeological observations

Springs discharge at various positions on talus cones and slopes (Fig. 1): SP1 and SP2 near the top, SP3 in mid-slope, and SP4 and SP5 near the bottom. This contrasts with most of previous studies on talus hydrology, which reported springs at the bottom of talus slopes (e.g. Caballero et al. 2002; Clow et al. 2003; Muir et al. 2011), suggesting the presence of different groundwater flow paths discharging at different positions.

Discharge from SP1 and SP2 was observed during the geophysical survey campaign in July 13–30, 2015, but not on October 22 during a field visit. Based on the location of these springs near the headwall and the short duration of flow, they are likely fed by the shallow groundwater flow sustained by late-season snowpack melting at the top of the talus and the headwall above. Discharge from SP3 persisted until October 22 and likely later into fall and winter. It is located directly downslope from a waterfall (Figs. 1 and 2), which flowed year-round as indicated by the presence of an icefall during winter site visits. Based on their relative locations and the persistence of flow, SP3 is likely sourced by the waterfall. The groundwater discharged from these top- and mid-slope springs flowed overland for a relatively short distance (20–30 m) along fluvial channels (like that pictured in Fig. S1d in the ESM) and disappeared into the talus deposits.

SP4 and SP5 are located at the bottom of talus deposits (Fig. 1), suggesting that these springs occur at the contact between a coarse permeable zone and a less permeable zone, consistent with previous studies elsewhere. The location of SP4 downslope of SP3 (Fig. 1) indicates a connection between the waterfall-SP3 system and SP4, whereas no spring was observed in the talus upslope of SP5. The groundwater discharged from SP4 and SP5 is the source of the West Meadow Stream (WMS) and the East Meadow Stream (EMS), respectively. The WMS flowed throughout summer and fall of 2015 and 2016, but the EMS dried up by mid-summer in both years.

**Fig. 4:**
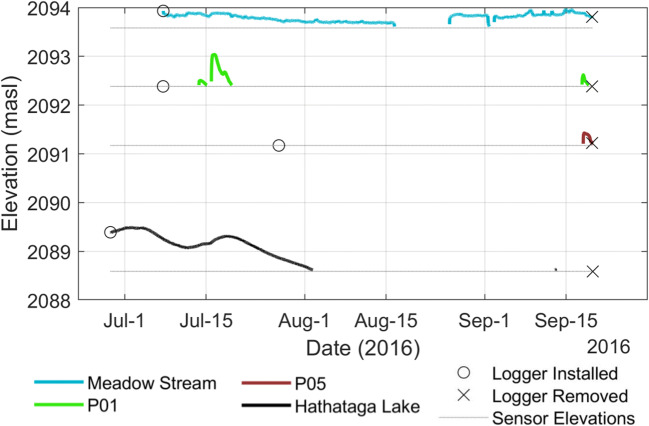
Piezometric data from 2016 collected in the meadow and lake. Dashed lines indicate the elevation of (and hence lower detection limit of) the sensors. The stream measurements are taken from the stilling well labelled “MSW” in Fig. 3

These streams incise up to 1 m below the meadow plain and flow over the fine-grained meadow sediments. The two streams meet in the north-central section of the meadow before flowing into Hathataga Lake. The water table under the meadow by stream channels was consistently lower than the stream water level (Fig. 4), indicating that these are losing streams. The flow in the merged meadow stream entering Hathataga Lake was 2.8 × 10^−3^ m^3^ s^−1^ on July 22 and 4.6 × 10^−4^ m^3^ s^−1^ on July 28, 2015. Dividing these values with the apparent drainage area of the meadow stream (0.8 km^2^) give a basin surface runoff in a range of 0.05–0.30 mm day^−1^. In comparison, total precipitation during July 1–28 was 96 mm, and much of the drainage area has no vegetation cover (Figs. 2 and 3), implying little evapotranspiration. Considering additional melt contribution from the late-lying snowpack in high-elevation areas, the magnitude of water input is expected to be on the order of 3 mm day^−1^. The relatively small magnitude of surface runoff suggests that the majority of water inputs to the talus-meadow complex from talus cones and slopes is drained by groundwater.

Combined groundwater inputs to Hathataga Lake from discharge points on the south shore (Fig. 1) was visibly greater than the surface water input from the meadow stream, although it was difficult to quantify the groundwater inputs. The water level in Hathataga Lake dropped by 0.8 m during July 13–29, 2015 (H. Wu, University of Saskatchewan, unpublished data, 2015), despite the surface and groundwater inputs, and rainfall input of 56 mm during this period. This indicates a large groundwater output (i.e. recharge) from the lake, implying that the lake water level is a surface expression of the water table in the moraine, similar to observations made elsewhere (e.g., Langston et al. 2013). The lake bed was exposed by August 11, and small streams originating from discharge points were flowing over the lake bed and terminating near the north end of the lake in a shallow (<15 cm) pond recharging groundwater. The groundwater recharged in Hathataga Lake and the talus-meadow area eventually discharged from a complex of several springs including SP6 and SP7 on the north side of the lake (Fig. 1), which is considered the outlet of the Hathataga Lake basin. The stream originating from the outlet sustained a steady baseflow throughout winter months of January–April (e.g., 0.008 m^3^ s^−1^ measured in February 2017) when there was little snowmelt or rainfall inputs, indicating a large storage capacity of the aquifer system.

### Hydrochemical and isotopic observations

**Fig. 5:**
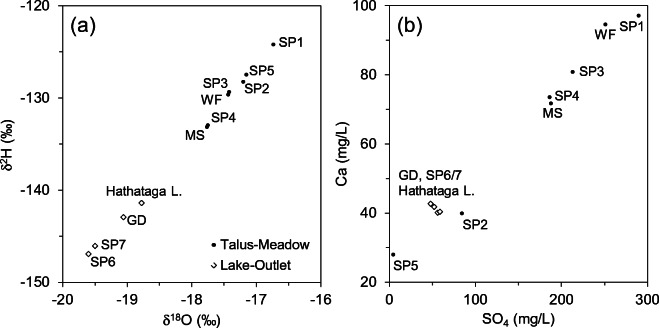
**a** Stable isotopic ratios of water samples from the springs (SP), the waterfall above the Central Cone (WF), the meadow stream (MS), one of the lakeshore groundwater discharge points (GD), and Hathataga Lake. **b** Dissolved Ca and SO_4_ concentrations of water samples. All samples were collected during July 14–28, 2015

Stable isotopic compositions of the waterfall, talus springs (SP1–SP5) and the meadow stream samples collected during July 14–28, 2015 had relatively heavy values (Fig. 5a) indicative of the influence of summer rainfall, which had a volume-weighted long-term (2006–2015) mean δ^18^O = −15.9‰ measured at the Barrier Lake Field Station located 27-km northeast of the study site (B. Mayer, University of Calgary, unpublished data). This suggests that the majority of water was sourced by summer rain, not snowmelt, implying a relatively short residence time of less than 1 or 2 months and shallow flow paths feeding the talus springs. In contrast, the samples from Hathataga Lake and a lake-shore groundwater discharge point (GD) had lighter values indicative of annual average precipitation composition (δ^18^O = −18.6‰, B. Mayer, University of Calgary, unpublished data, 2019). This supports the idea that most of water in Hathataga Lake is sourced by groundwater. It also suggests that groundwater in the aquifer connected to Hathataga Lake has a longer residence time (1 year or more) than the talus springs and, thus, is part of the deeper flow system.

All water samples had relatively low total dissolved solids ranging from 120 to 520 mg L^−1^. The variability is characterized by Ca and SO_4_ concentrations (Fig. 5b), indicating the influence of CaSO_4_ inputs to SP1, the waterfall-SP3–SP4 system, and the meadow stream fed by SP4. Hathataga Lake, GD, and SP6/SP7 had much smaller concentrations implying a separate flow system consistent with the isotope signatures (Fig. 5a). SP2 and SP5 also had low concentrations indicating that the recharge sources of these springs are different from SP1–SP4. The source of Ca and SO_4_ is likely the oxidation of iron sulfide minerals (e.g. pyrite) commonly contained in shale layers such as those in the Exshaw Formation exposed near the top of the headwall (Fig. 1).

Based on the hydrogeological and hydrochemical data, the occurrence of talus springs appears to be controlled by the internal structure of talus deposits, and there are shallow and deep groundwater systems connecting the talus-meadow complex to the basin outlet springs. Geophysical imaging techniques are used to examine how the spring locations are controlled by geological structure and how the shallow and deep groundwater systems are connected to form the alpine aquifer system.

### Geophysical imaging of talus cones and slopes

**Fig. 6:**
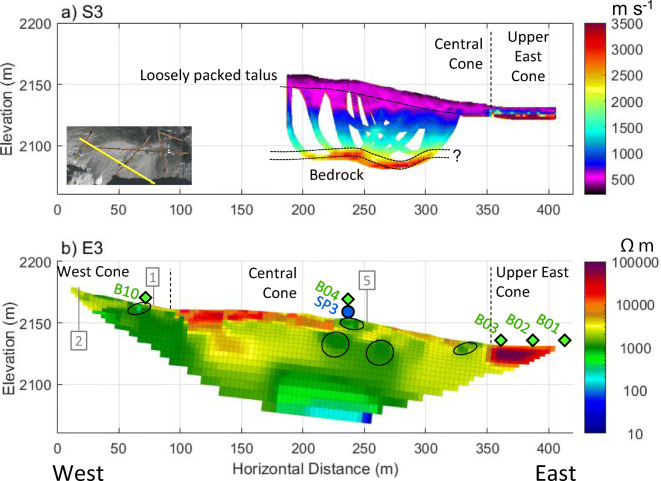
Geophysical Line 3 crossing West Cone and Central Cone. **a** P-wave velocity model from S3, with RMS = 2.1 ms. **b** Electrical resistivity model from E3, with an absolute error of 6.2%. Annotations include: tie points with other lines as numbered in Fig. 3 (grey, numbered boxes), springs (blue circle), BTS sensors (green diamonds), boundaries between neighbouring talus cones (vertical dashed lines), and areas of concentrated groundwater flow (ellipses with solid black outline). Data gaps in the layer above bedrock (**a**) are due to limited ray path coverage. The exact geometry of the bedrock surface is somewhat uncertain, as expressed by the two dotted lines (**a**)

Geophysical surveys were conducted on five lines over talus cones and slopes (Fig. 3) to investigate the influence of internal structure on groundwater flow paths. Line 3 traversed the mid-slope position of talus cones and showed three layers of P-wave velocity (Fig. 6a). The choice of three-layer interpretation is based on the convergence of ray paths to these three elevations (Fig. S5 of the ESM). The upper 10–20 m of the Central Cone had velocities below 500 m s^−1^, indicating a mantle of loose unconsolidated sediments. The middle layer, which has velocities ranging between 500 and 1,500 m s^−1^, consists of more densely packed talus where air gaps between large clasts have been filled in by finer grains. This is consistent with other studies, which found similar mantling of looser material in the upper portion of talus deposits (Sass 2006; Götz et al. 2013).

The bottom layer had velocities between 2,300 and 3,500 m s^−1^ and is interpreted to be bedrock. The sharpness and exact position of the bedrock surface along Line 3 (Fig. 6a) is difficult to determine given its large depth, the relatively low density of ray paths crossing it, and some uncertainty in the travel-time picks due to the low energy of the sledgehammer source. Supplementary data from the eastern ridge of the Hathataga Lake Basin further supports the bedrock velocity interpretation. Line 12 crosses outcrops of both the Palliser and Fernie Formations (Fig. S6 of the ESM). Unweathered and intact Palliser and Fernie Formations had P wave velocities of 3,500–5,000 m s^−1^ and 2,500–3,800 m s^−1^, respectively. However, at these locations, the Palliser Formation was lightly fractured at surface, and the Fernie Formation outcrop was highly fissile, weathered shale. While a velocity of 2,300 m s^−1^ may seem too low to interpret as a bedrock boundary, other studies have found that in alpine zones, even light fracturing can significantly reduce the seismic velocity of bedrock (e.g. McClymont et al. 2011). Hence, between intact rock and the talus, there is likely an upper layer of fractured bedrock, though a thin layer of stiff sediments like till cannot be entirely ruled out.

**Fig. 7:**
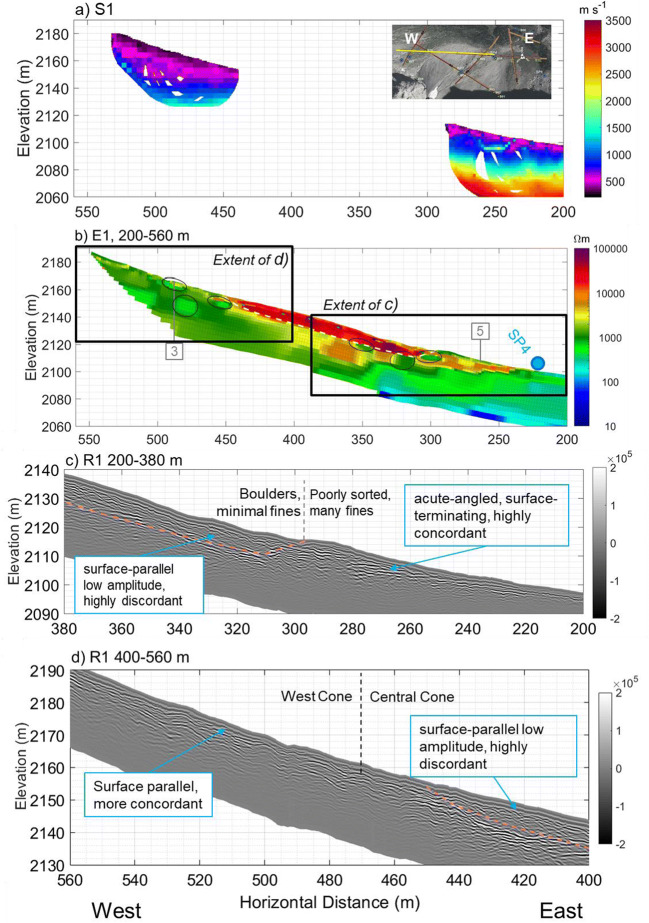
**a** P-wave velocity models (RMS = 1.8 ms) and **b** resistivity model (absolute error = 4.8%) along the portion of Line 1 crossing talus deposits. Annotations indicate the locations of: springs (blue circle), tie points with other lines as numbered in Fig. 3 (grey, numbered boxes), and convergent flow paths (black ellipses). A sample of GPR data showing the differences in texture near the base of the Central Cone (**c**) and between the west and Central Cone (**d**)

**Fig. 8:**
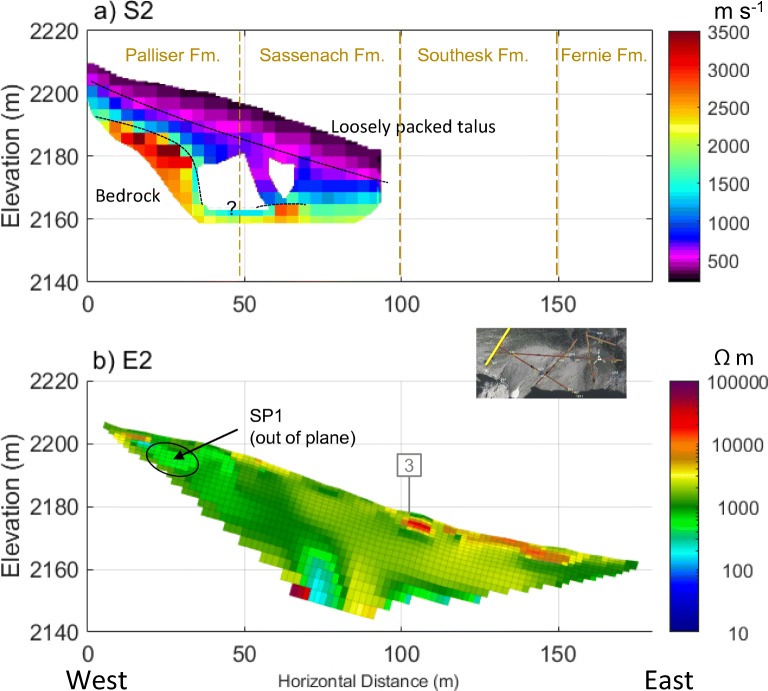
Composite of the **a** P-wave velocity model (RMS = 2.6 ms) and **b** resistivity model (absolute error = 3.2%) along line 2. Annotations indicate the locations of: tie points with Line 3 at approximately 100 m (grey box with “3”), convergent flow paths (black ellipses), and the locations of inferred geological contacts from McMechan (2012) (dashed tan lines). Data gaps in the layer above bedrock (**a**) are due to limited ray path coverage

**Fig. 9:**
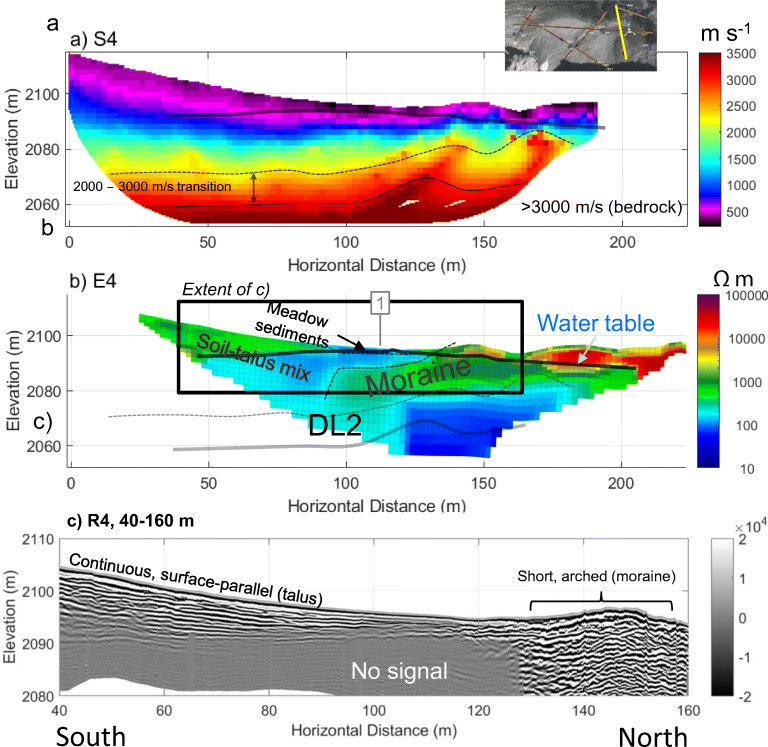
Geophysical images crossing South to North across the meadow: **a** P-wave velocity model along S4 (RMS = 1.4 ms); **b** electrical resistivity model along E4 (absolute error = 3.3%); **c** radar reflection image along R4. Black solid and dashed lines (**a**–**b**) indicate boundaries interpreted from that data set whereas semi-transparent lines indicate boundaries interpreted from other lines. Grey box with “1” at approximately 110 m indicates tie point with Line 1. See Fig. 3 for line locations

A similar three-layer structure of P-wave velocity like that of line 3 was observed in line 2 (Fig. 8a), and line 4 (Fig. 9a). The thickness of talus was up to 60 m in mid-slope (Fig. 6a, 200–300 m) and appeared to decrease to 20–30 m in the lower position (Fig. 7a, 200–250 m). Closer to the top of the talus cones, near the headwall, bedrock had a “step-down” feature, where the bedrock elevation abruptly decreased from within 10–15 m of the talus surface to 30 m (Fig. 8a, 10–30 m). Similar stepped bedrock topography has been observed elsewhere (Sass and Krautblatter 2007; Muir et al. 2011; Götz et al. 2013; Volze 2015; Brody et al. 2015).

Resistivity results in all survey lines (Figs. 6b, 7b, 8b, 9b, and Fig. S6 of the ESM) showed three groups of material: high resistivity (15,000–65,000 Ωm, red in the ERT images), intermediate resistivity (3,000–10,000 Ωm; yellow or orange), and low resistivity (500–3,000 Ωm; light green to dark green). Visual observations of the three groups at or near the talus surface showed that the high resistivity group correlated with areas underlain by boulders with minimal fine sediments and high porosity (e.g. Fig. 7b, 300–450 m). The intermediate resistivity group corresponded with talus with gaps between coarse rubble filled with fine-grained sediments and with increased vegetation covers (e.g. Fig. 7b, 250–300 m). The low resistivity group was similar to the intermediate resistivity group, but it corresponded with wet sediments near or downslope of springs (e.g. Fig. 6b, 230–240 m), or with a greater fraction of fine-grained sediments (e.g. Fig. 7b, 450–500 m). It also often corresponded to microtopography features like relict channels (Fig. 5b, 340 m).

The resistivity groupings are consistent with previous interpretations of resistivity (Hauck and Kneisel 2008; McClymont et al. 2010; Muir et al. 2011), and the geometry is also consistent with current understanding of how talus deposits form. Porosity of talus deposits generally decreases with depth because finer grains are carried down by water through pore space of the coarse boulders (White 1981; Sass 2006; Sass and Krautblatter 2007). Hence, coarser material is expected near the surface, and indeed, the highest-resistivity unit occurs only in the upper 10–15 m of the talus. All resistivity images showed discontinuous bodies of different resistivity units within the thick talus deposits (annotated in figures), suggesting the complex history of talus formation, which lead to a complex geometry of low-permeability layers and higher permeability lenses present together in the same slope. This is consistent with observations of such contrasts at surface (Fig. S1d of the ESM) and with earlier geomorphological studies of alluvial talus (van Steijn et al. 1995).

Ground-penetrating radar radargrams showed rudimentary bedding throughout the talus (Figs. 7c,d and 9c), but with differing textures. Areas that had lower-resistivity surface cover and lied downstream of the waterfall on the Central Cone tended to have more concordant reflectors that terminate at the surface with an acute angle (e.g. Fig. 7c, 230–290 m). Elsewhere, reflectors were surface-parallel and discordant (Fig. 7c,d, 300–450 m). Several authors have argued that talus deposits formed by different processes have distinct and diagnostic GPR textures (Sass 2006, 2007; Sass and Krautblatter 2007; Onaca et al. 2016). Based on those results, areas below the waterfall, which have surface-terminating, concordant reflectors, are composed of alluvial talus. Similarly, other areas with weaker, surface-parallel, discordant reflectors are interpreted as rockfall talus.

Three talus springs (SP1, SP3, and SP4) are located on or near the survey lines (Fig. 3). The bedrock surface under these springs was at least 20 m below the discharge points, clearly indicating that their locations are not controlled by bedrock as commonly reported elsewhere (Caballero et al. 2002; Clow et al. 2003; Muir et al. 2011). Based on the presence of higher resistivity under the low resistivity, saturated zone at SP3 (Fig. 6b), it is likely that SP3 is part of a shallow, perched groundwater flow system recharged by the waterfall. A similar condition may apply to SP1.

The east end of line 3 is in the shaded area that receives direct solar radiation only for a few hours even in midsummer (Fig. 3). The high resistivity (65,000–75,000 Ωm, see Fig. 6b) and P-wave velocity (3,500–5,500 m s^−1^, see Fig. 6a) are indicative of permafrost consisting of rock-ice mixture (Hauck and Kneisel 2008). The bottom temperature of snowpack (BTS) ranged between −3.9 and − 3.0 °C for the sensors located in this section (B01, B02, B03), indicating “probable permafrost” (Hoelzle et al. 1999). In contrast the BTS at other locations on the same line (B04, B10) ranged between −0.6 and − 0.1 °C, indicating “improbable permafrost”. Therefore, all data sets are consistent with permafrost in the shaded section of line 3, implying that permafrost may be present in this and similar alpine watersheds in the region when conditions are favourable.

### Geophysical imaging of meadow sediments and moraine

**Fig. 10:**
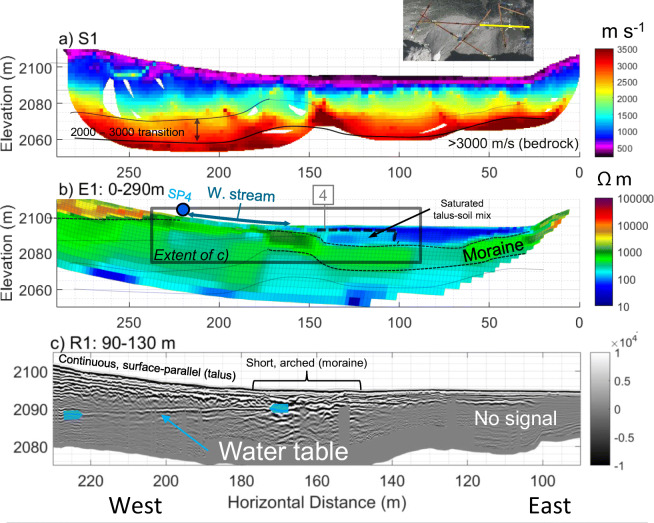
Geophysical images crossing west to east across the meadow: **a** P-wave velocity model (RMS = 1.8 ms) along S1 (east segment); **b** electrical resistivity model from E1 (absolute error = 4.8%); **c** radar reflection image from R1. Black solid and dashed lines (**a**–**b**) indicate boundaries interpreted from that data set, whereas semi-transparent lines indicate boundaries interpreted from the other geophysical models. Other annotations include: tie points with line 4 at approximately 140 m (grey box with “4”) and springs (blue circle). See Fig. 3 for line locations

**Fig. 11:**
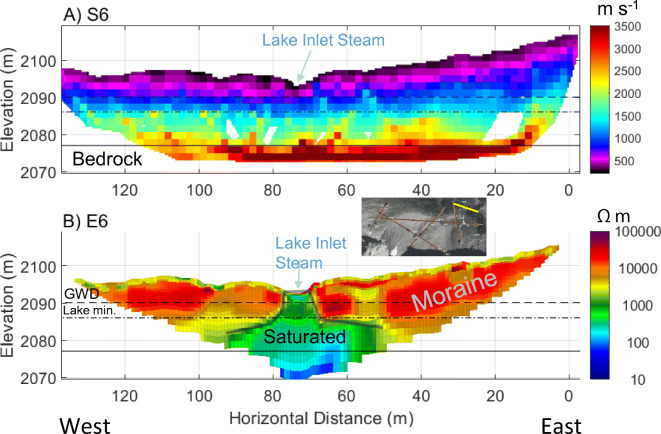
Geophysical models from the moraine along the south side of Hathataga Lake: **a** P-wave velocity model from S6 (RMS = 3.5 ms); **b** electrical resistivity model from ERT6 (absolute error = 9.9%). Annotations note the elevation of groundwater discharge points (GWD) on the south shore of Hathataga Lake (dashed black line), the minimum elevation of the lakebed (dot-dashed line), the interpreted depth to bedrock (solid black line), and the location of the inlet stream from the meadow to the lake (blue arrow), and the saturated zone below the lake inlet (semi-transparent solid line). See Fig. 3 for line locations

Generally, seismic images showed that the material near the meadow surface had a P-wave velocity of 300–500 m s^−1^, with lower velocity in the centre of the meadow (Figs. 9a and 10a). In between the low-velocity surface layer and bedrock was a transition zone of sediments with the velocity gradually increasing with depth. A high-velocity (>2,000 m s^−1^) layer was present at depth throughout the meadow and the moraine. As discussed in the section “Geophysical imaging of talus cones and slopes”, this is somewhat lower than the velocities observed for intact rock along at line 12 (Fig. S6 of the ESM). The transition from a relatively low (~2,000 m s^−1^) to a high velocity (>3,000 m s^−1^) suggests that this layer is bedrock with a substantial fractured and weathered zone near the bedrock surface. This is highly plausible given that the Fernie Formation, which is mapped at this location (Fig. 1), is highly fissile at some outcrop locations in the area. Without borehole confirmation, the 2,000–3,000 m s^−1^ layer could still be plausibly interpreted as stiff sediments such as a till. Assuming that the top of bedrock is at around 2,000 m s^−1^, bedrock elevation changed from ca. 2,070 masl below the meadow (Figs. 9a and 10a) to 2,080 masl at the south shore of Hathataga Lake (Fig. 11a).

The resistivity images showed a complex arrangement of different resistivity units. The moraine on the south shore of Hathataga Lake had the characteristic high resistivity (>3,000 Ωm) except for a thin layer of top soil (Fig. 11b). The moraine resistivity was much lower (<2,000 Ωm) in the mound-shaped saturated zone formed by the infiltration of stream water (50–90 m), and even lower in bedrock suggesting the presence of a deep groundwater system connecting the meadow and Hathataga Lake through the coarse moraine and weathered shale.

The saturated moraine extended from the northern edge of the meadow to the centre, where it was overlain by a low-resistivity (30–80 Ωm) layer (Fig. 9b, 90–130 m and Fig. 10b, 30–140 m). The thickness of this low-resistivity layer was ca. 10 m in the meadow centre. Based on sediment samples collected by hand augering, this fine-grained sediment is interpreted to be of lacustrine origin. Rock fragments are found when auguring occasionally, though these were likely transported by avalanche as observed during winter site visits. The moraine appeared to terminate on the south side of the meadow (Fig. 9a, 90 m), and the thickness of lacustrine sediments increased to ca. 20 m. This layer was covered by boulders extending from the Lower East Cone. The boulders appeared to contain fine-grained sediments based on relatively low resistivities (<700 Ωm). The GPR images give little information about deeper portions of the meadow, especially below the most conductive portions of the meadow sediments where no signal was obtained (Fig. 10b, 30–100 m). However, the radargrams in Figs. 9c and 10c show that talus and moraine sediments had different textures: the talus had continuous, defined layering and the moraine had chaotic, arched reflectors.

The GPR and ERT images indicate a flat reflector at 2,091–2,092 masl occurring below the transition region between the meadow and East Cone (Figs. 10, 180–210 m). No GPR signal was detected below this level, thus indicating a wet condition. This reflector coincided with the reduction in resistivity from ~700 to 150 Ωm (Fig. 10b, 50–100 m). The change in resistivity is of a much lower magnitude than other locations on the study site but is still visible when using a slightly narrowed coloured scale in Fig. S7 of the ESM. Given that the small pore spaces between fine grains leads to capillary rise, the magnitude of the water content change and the sharpness of the resistivity boundary are expected to be subdued. There was no measurement of the water table under the meadow during the geophysical data acquisition in July 2015, but measurements during July–September 2016 indicated the water table lower than 2,092 m most of the season (Fig. 4). Therefore, the GPR reflector and the resistivity boundary at 2,091–2,092 masl is interpreted to be from within the capillary fringe near the water table, which is located 4–5 m below the discharge point of SP4 (Fig. 10b). This suggests that the shallow groundwater flow system discharging at SP4 is perched above the deeper groundwater system under the meadow.

### Geophysical imaging of the basin outlet

**Fig. 12:**
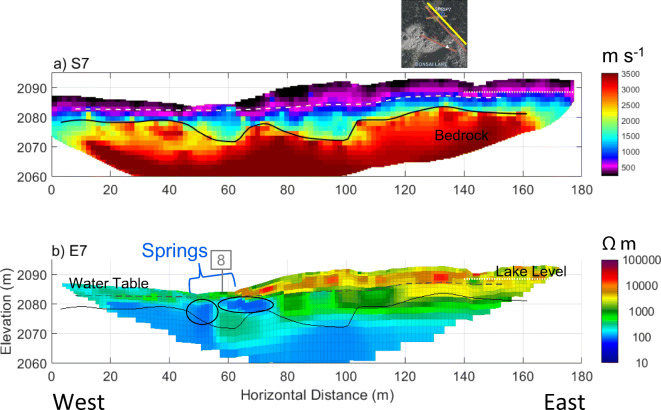
Geophysical models near the northern outlet spring: **a** along S7 (RMS = 4.5 ms) and **b** E7 (absolute error = 9.5%). Annotations note: the elevation of the interpreted depth to bedrock (solid black line), the interpreted depth to saturation (grey-dashed line), the location of the outlet springs SP6 and SP7, the tie-point with line 8 at approximately 60 m (grey box with “8”), low-resistivity anomalies at depth (black ellipses), and the elevation of Hathataga Lake (2,088.5 masl) at the time of survey (white-dotted line). See Fig. 3 for line locations

Seismic images on the north side of Hathataga Lake (Fig. 12a) showed a high velocity (>2,000 m s^−1^) layer at ca. 2,080 masl, which is interpreted to be the shale unit of the Fernie Formation, based on outcrop observations directly north of the outlet spring complex. The bedrock surface at this location occurred at a similar elevation as the bedrock surface on the south side of the lake (Fig. 11a). There were substantial variations in bedrock topography, although the exact shape of these was poorly constrained because of the low signal to noise ratio in seismic signals. The bedrock appeared to have two depressions centered at 60 and 95 m (Fig. 12a), with the former being located directly below the outlet spring complex.

The upper part of the moraine had a high resistivity (3,000–20,000 Ωm) similar to the moraine on the south side of Hathataga Lake. The lower part below the Hathataga Lake water level (2,088–2,089 masl) had lower resistivity suggesting the presence of the water table within the moraine (Fig. 11b). Similar drop in resistivity at the same elevation was observed in other lines on north of the lake (Figs. S8–S10 in the ESM). These images showed anomalies with resistivities under 100 Ωm, many of which corresponded with the bedrock depressions (e.g. Fig. 12a, 60–70 m), suggesting the possibility of relatively narrow groundwater flow paths within the moraine above the bedrock surface.

## Discussion

### Hydrogeological conceptual model

**Fig. 13:**
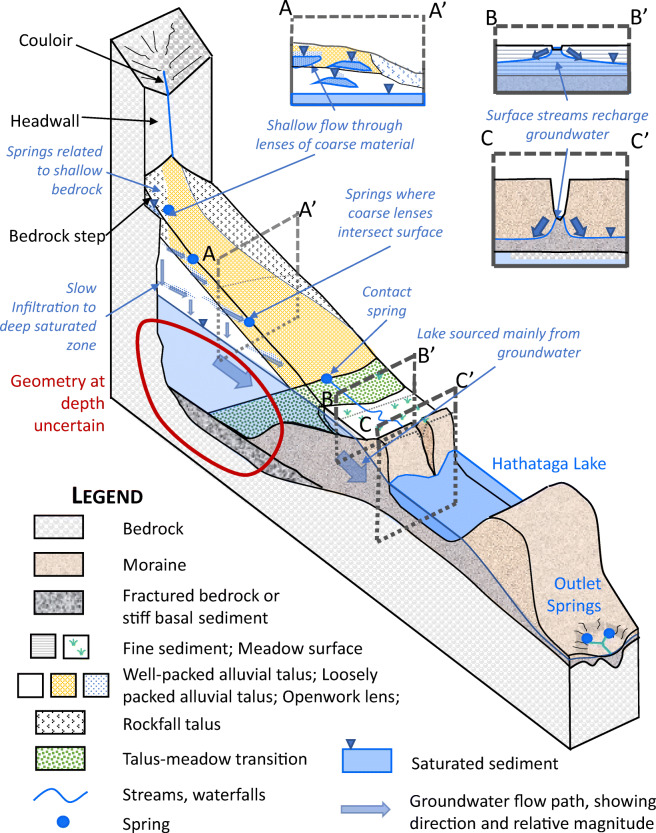
Conceptual model of subsurface geology and flow paths. Drawing is schematic and not to scale

The elevation of the bedrock surface rises by ca. 10 m from under the meadow to the moraine encircling Hathataga Lake based on the seismic data (Figs. 9a, 10a, 11a), suggesting that the meadow lies within a post-glacially filled glacial overdeepening (alternatively called a subglacial basin). This has led to an accumulation of sediment up to 30–40 m in the meadow as schematically shown in Fig. 13. This geometry resembles that of other overdeepenings reported in the literature. In their review of bedrock-scoured basins, Cook and Swift (2012; Fig. 3) put forth three general models that capture the variety of morphologies observed, one of which is partly confined by a terminal moraine, which is consistent with this case. Moreover, the length (200–400 m) and depth (30–40 m) of this basin are within the range of basins reviewed by Haeberli et al. (2016).

The thick sediment packages consisting of moraine, talus, and meadow-fill form the interconnected alpine aquifer system of the Hathataga Lake basin (Fig. 13). Exposed bedrock of headwalls, occupying ~40% of the watershed (Fig. 1), has limited infiltration capacity and forces most of snowmelt and rain water to flow over the rock surface and enter the top of talus cones and slopes. Couloirs in the headwalls focus the melt water from late-lying snowpack and small amounts of seepage from fractured bedrock to the waterfalls above talus cones (Fig. 2). The high SO_4_ concentration in the waterfalls (Fig. 5b) indicates that the water is either coming through fractures in the pyrite-containing shale unit of the Exshaw Formation (Fig. 1) or through the shale-derived sediments accumulating in the couloir.

There are two distinct shallow and deep groundwater systems present. Geochemical data show that the waterfall, SP3, and SP4 all have similar chemical and isotopic compositions that are distinct from other features (Fig. 5b). The shallow groundwater discharging from SP4 and SP5 flows through the meadow streams, which lose water to underlying sediments due to the high hydraulic gradient indicated by the piezometric data (Fig. 4).

Separate shallow and deep flow systems in talus are not unusual in talus (Davinroy 2000; Roy and Hayashi 2009), but the springs on the talus at Hathataga are uncommon. Sediment heterogeneity explains their occurrence. Whereas some areas of the talus only have a mantle of uniform, blocky rockfall material in the upper 15 m (Figs. 7, 200–450 m), springs occur where there is a mix of fine-grained layers and coarser layers or lenses (e.g. Fig. 6b, 230–240 m). These fine-grained layers slow down infiltration, and contiguous bodies of coarse-grained sediments quickly convey water laterally along the slope. These springs therefore occur where the coarser layer insect the surface (Fig. 13).

The deeper groundwater in talus units flows into the meadow and moraine, and discharges at the south shore of Hathataga Lake. Slopes on the west and east side of the moraine consist of the bedrock of the Fernie Formation with a relatively thin (<2–3 m) soil cover based on visual observations. As a result, the deep groundwater is forced to flow through the moraine and discharge at the outlet spring complex. The interconnected aquifer system consisting of unconsolidated sediments plays an important role in storing snowmelt and rain water and releasing it over a long time and maintaining the streamflow throughout the year. The outlet spring complex can be seen as the “gate keeper” of the alpine headwater basin. Similar gate-keeper functions have been reported for headwater springs discharging from coarse blocky sediments such as moraines (e.g., Roy and Hayashi 2009) and rock glaciers (e.g., Winkler et al. 2016) elsewhere in the world. At the Hathataga Lake basin, heterogeneous sediments consisting of high- and low-conductivity materials likely enhance the storage capacity and internal connectivity of the aquifer system. This is consistent with previous findings that reported the importance of high-conductivity sediments in connecting talus slopes to streams (Baraer et al. 2014; Gordon et al. 2015) and the importance of low-conductivity sediments in storing water and buffering seasonal variations in freshwater supplies (Clow et al. 2003; Roy and Hayashi 2007; Weekes et al. 2015; Fischer et al. 2015).

Much of the talus-meadow-moraine aquifer system is underlain by the shale unit of the Fernie Formation (Fig. 1), which is intensely fractured at outcrops exposed on the eastern ridge of the watershed and along the outlet stream. The seismic data indicate that the upper bedrock is fractured and/or weathered under the meadow (Figs. 9a and 10a), though alternative explanations like a basal till cannot entirely be ruled out. A significant zone of fractured shale may contribute additional storage capacity and connectivity to the aquifer system. This contrasts with findings from past studies at the Lake O’Hara basin in the Main Range of the Canadian Rockies, located 100 km northwest of Hathataga Lake. That basin is underlain by much more competent Cambrian quartzite rocks (Price et al. 1980), and fractured rocks played an insignificant role in the water balance (Hood and Hayashi 2015). Comparison of the two contrasting sites in proximity to one another highlights the influence of bedrock geology on the hydrogeology of the aquifer system derived from the bedrock.

### Geomorphological implications

In this study, geophysical imaging has helped detect two examples of infrequently observed landforms: an overdeepened subglacial basin and discontinuous permafrost in talus. In particular, overdeepened basins are infrequently documented because they are usually obscured by ice or post-glacial sediments (Cook and Swift 2012; Haeberli et al. 2016). These additional data points will assist ongoing studies on the distribution and formation of these features.

While this study has established that talus slope hydrology at Hathataga *is* different from past studies, it is natural to ask *why* it is different. A direct comparison with the study of Muir et al. (2011) at the Lake O’Hara basin hints at potential causes. Higher rockfall rates are expected at the Hathataga Lake basin for several reasons: the headwall consists of weaker and younger bedrock (Bieniawski 1974, 1989; Moore et al. 2009), has a more shaded aspect (Gardner 1983; Sass and Wollny 2001; Sass 2007), and is a large thrust fault scarp (Butler et al. 1986; Coe and Harp 2007), which may explain the thicker talus deposits at Hathataga. Similarly, Hathataga’s headwall has much more pronounced couloirs, so more redistribution of sediment by fluvial processes is expected (Fryxell and Horberg 1943; van Steijn et al. 1995; Sass and Krautblatter 2007), which would in turn lead to a denser packing of talus with more fine grains (White 1981; de Scally and Owens 2005). This is consistent with observations: talus at Lake O’Hara lacks alluvial talus features described by White (1981) and has less fine-grained material. It is these two key differences—the thickness and packing—that leads to a very different hydrological regime in talus at Hathataga. Although this site-to-site comparison is far from rigorous, it points to plausible causal links between geomorphological processes, lithology, and alpine hydrogeology.

More rigorous study of the linkage between geomorphology and hydrogeology is merited given the benefits that may result from such work. One of these may be better methods for transferring hydrogeological conceptual models based on local-scale studies (e.g. Fig. 13) to regional scales. With enough case studies, it may be possible to, for example, predict which set of hydrogeological regimes are plausible based on geospatial data such as geological maps, digital elevation models, and aerial images, thereby improving the performance of regional hydrological models. Statistical studies between geomorphic variables and hydrological variables like Paznekas and Hayashi (2016) have only had limited success at advancing towards this goal because their scale of investigation (200–4,000 km^2^) was too large to detect most correlations between geomorphic variables and hydrological variables. Therefore, further studies should consider a variety of alpine regions characterized by different geology and climate to develop a broader range of hydrogeological conceptual models. They should also focus on linking the observation of geomorphic variables and processes in small basins (<15 km^2^) to integrated behaviour of larger river basins using geospatial data analysis.

## Conclusions

The alpine cirque in this study, called Hathataga Lake basin, has a large groundwater storage capacity in the aquifer system consisting of interconnected sediment packages associated with typical alpine landforms, namely talus, moraine and alpine meadow. A conceptual hydrogeological model of the interconnected aquifer system (Fig. 13) has been established using hydrogeological observation, hydrochemical data, and geophysical imaging. The upper portion of the aquifer system consists of talus cones and slopes that receive snowmelt and rain water flowing from the headwall above and transmit it to the lower portion of the aquifer. Talus units are up to 60 m thick and spatially heterogeneous, reflecting the variability in source rocks and sediment redistribution processes. As a result, shallow groundwater flow paths develop over densely packed fluvial talus sediments and provide relatively fast pathways of groundwater from the waterfalls pouring out of couloirs in the headwall to mid-slope springs, and to talus-bottom springs. A shaded section of talus contains ground ice, indicating the presence of localized areas that can maintain permafrost under the present climate (or at least slow the thawing thereof). The shallow groundwater system is perched above the deeper groundwater system residing within the thick package of meadow sediments in the bedrock basin formed by glacial overdeepening. The deep groundwater flows from talus and meadow sediments to the moraine encircling Hathataga Lake, which is a surface expression of the water table, and eventually discharges to the spring complex at the basin outlet. The moraine is blocking a bedrock depression formed by a Pleistocene glacier and serving as a gate keeper of hydrological output from the cirque. The lower part of the basin is underlain by shale, which may have a zone of intense fractures and weathering near the surface. The fractured zone in the shale may provide additional storage and transmission functions to the aquifer system.

The landforms examined in this study are common features in mountainous regions around the world, and some aspects of the hydrogeological conceptual model are expected to be transferrable to other alpine basins. However, hydrogeological functions of talus in Hathataga Lake basin are substantially different from those of talus in previous studies, mostly because of the difference in the physical properties of bedrock producing talus sediments, and geomorphic processes (e.g. rockfall vs. fluvial erosion). Therefore, further field studies in a variety of locations will be needed to improve our understanding of how geological setting and geomorphic processes affect the hydrological characteristics of alpine land forms.

## Electronic supplementary material

ESM 1 (PDF 1.81 mb)
