# Supplementary material for: Hydrogeological characterization of an alpine aquifer system in the Canadian Rocky Mountains
Source: Hydrogeol J. 2020 May 4;28(5):1871–90. doi: 10.1007/s10040-020-02153-7 (PMC7373154; doi:10.1007/s10040-020-02153-7)
Supplement: Supplementary file 1 — (PDF 1.81 mb) [file 10040_2020_2153_MOESM1_ESM.pdf]

Hydrogeological characterization of an alpine aquifer system in the Canadian Rocky Mountains

Electronic Supplementary Material – Hydrogeology Journal

Craig W. Christensen[1,2†], Masaki Hayashi[1*], Laurence R. Bentley[1]

1: Department of Geoscience, University of Calgary, Calgary, Alberta, Canada

2: Norwegian Geotechnical Institute, Oslo, Norway

Email: †cch@emeraldgeo.no, *hayashi@ucalgary.ca

Contents

Supplementary Figures 2

Supplementary Tables 9

Appendix: Inversion Equations 11

References 13

Supplementary Figures

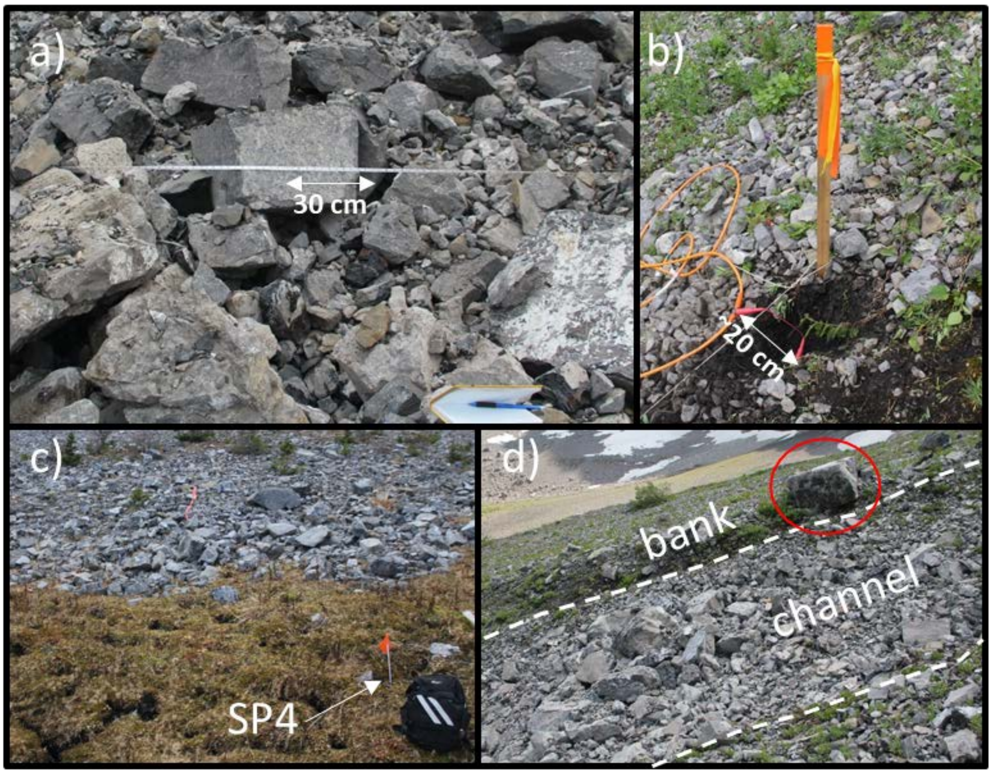

Figure S1: A sample of photos highlighting the differences in grain sizes and packing on the talus cones within the study area. (a) Large, loosely packed boulders with little infill on the eastern side of the Central Cone, located at approximately 375 m on E3. (b) Small cobbles and coarse gravel filled in with soil at the start of E2 on the West Cone. (c) A soil-talus mix at the fringe of the Central Cone near SP4 (backpack for scale). (d) Soil-talus mix on the west half of the Central Cone near the apex, a channel depression with minimal fine-grained sediments, loose packing, and less vegetation than the surrounding banks. Photos were taken on (a) July 22, (b) July 20, (c) October 22, and (d) July 8, all in 2015.

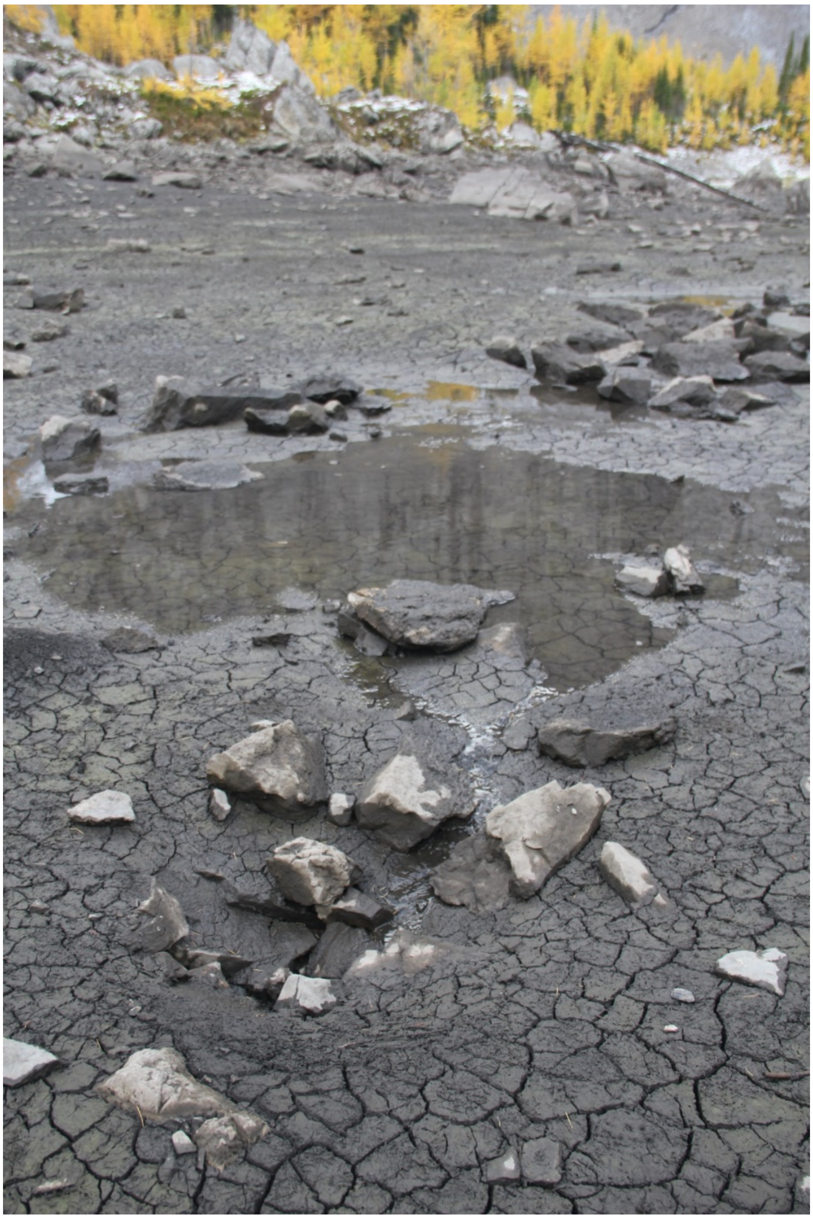

Figure S2: Field photo from September 19, 2016 showing standing water in the otherwise dry lakebed being discharged to underlying, coarse-grained sediments via an opening in the fine-grained sediments of the lakebed

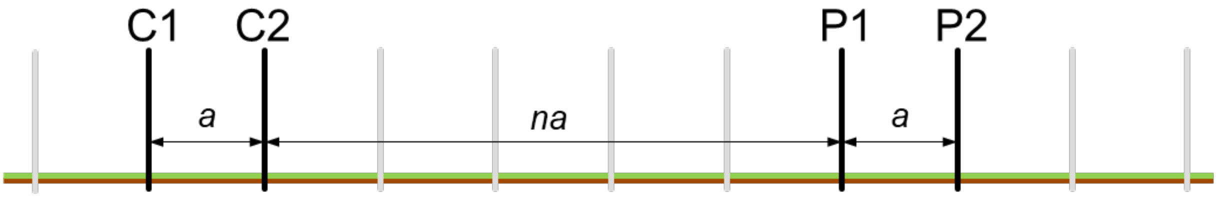

Figure S3: Schematic diagram illustrating the dipole-dipole array used in electrical resistivity tomography. Pairs of current injection electrodes (C1, C2) and potential measurement electrodes (P1, P2) spaced *na* apart are used in an array of electrodes with nominal spacing of *a*. In this study, *n* ranged from 1 to 6.

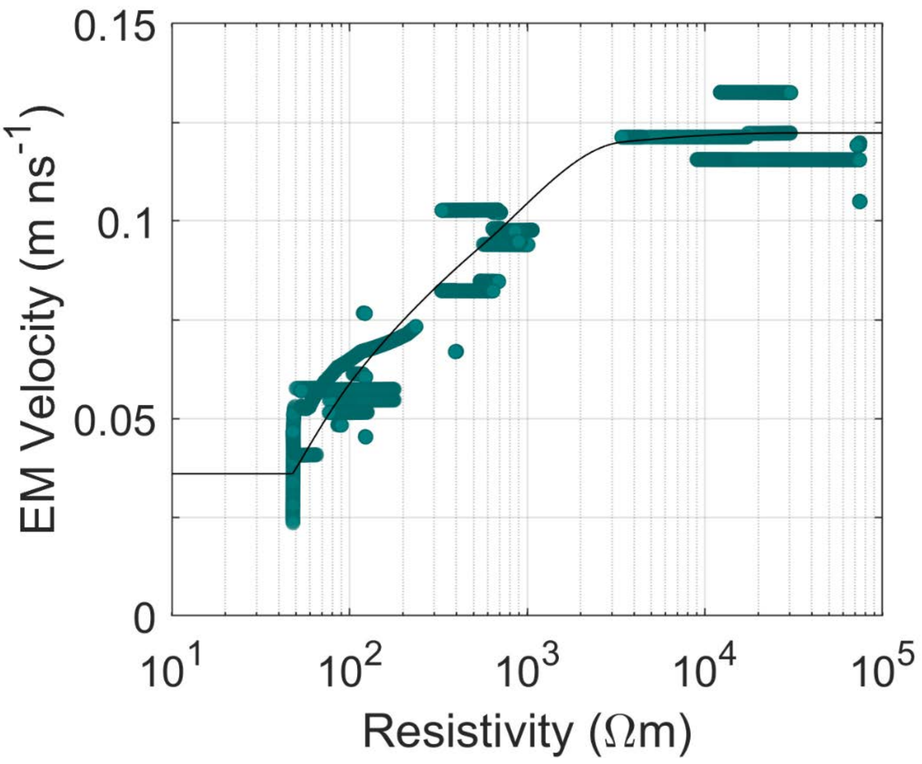

Figure S4 The correlation model (black line) used to estimate 2D EM-velocity distribution and in turn convert GPR reflection sections from time domain to depth domain. Data points above are collocated pairs of EM-velocity (from 1D-models resulting from semblance analysis) and electrical resistivity (interpolated from ERT images to CMP locations).

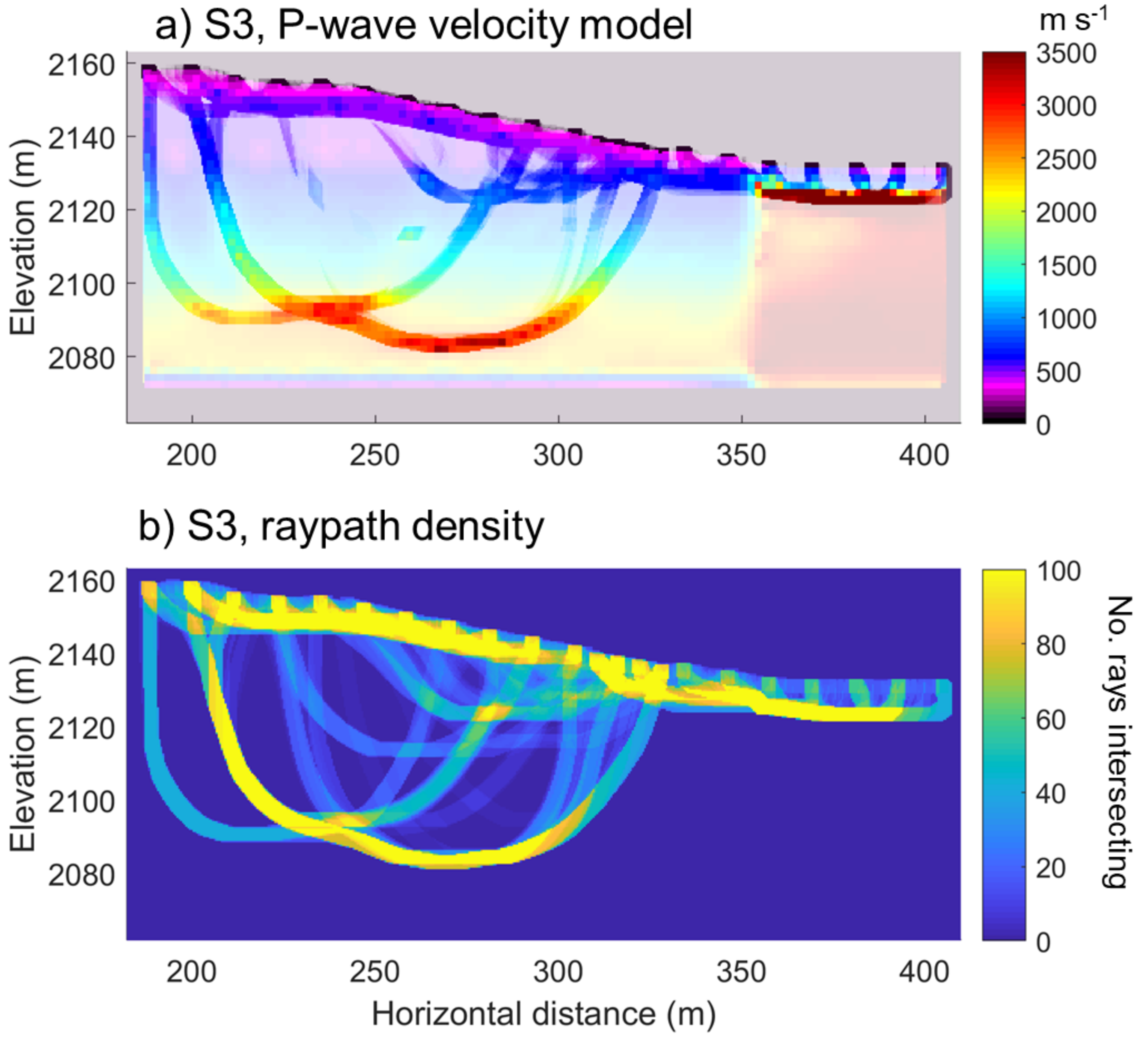

Figure S5 Seismic refraction tomography results for line S4: (a) the uncropped P-wave velocity model showing a transparency overlay for the raypath density; (b) the raypath density associated with this model showing a reflector within 10 m of surface, and a deep reflector below 2100.

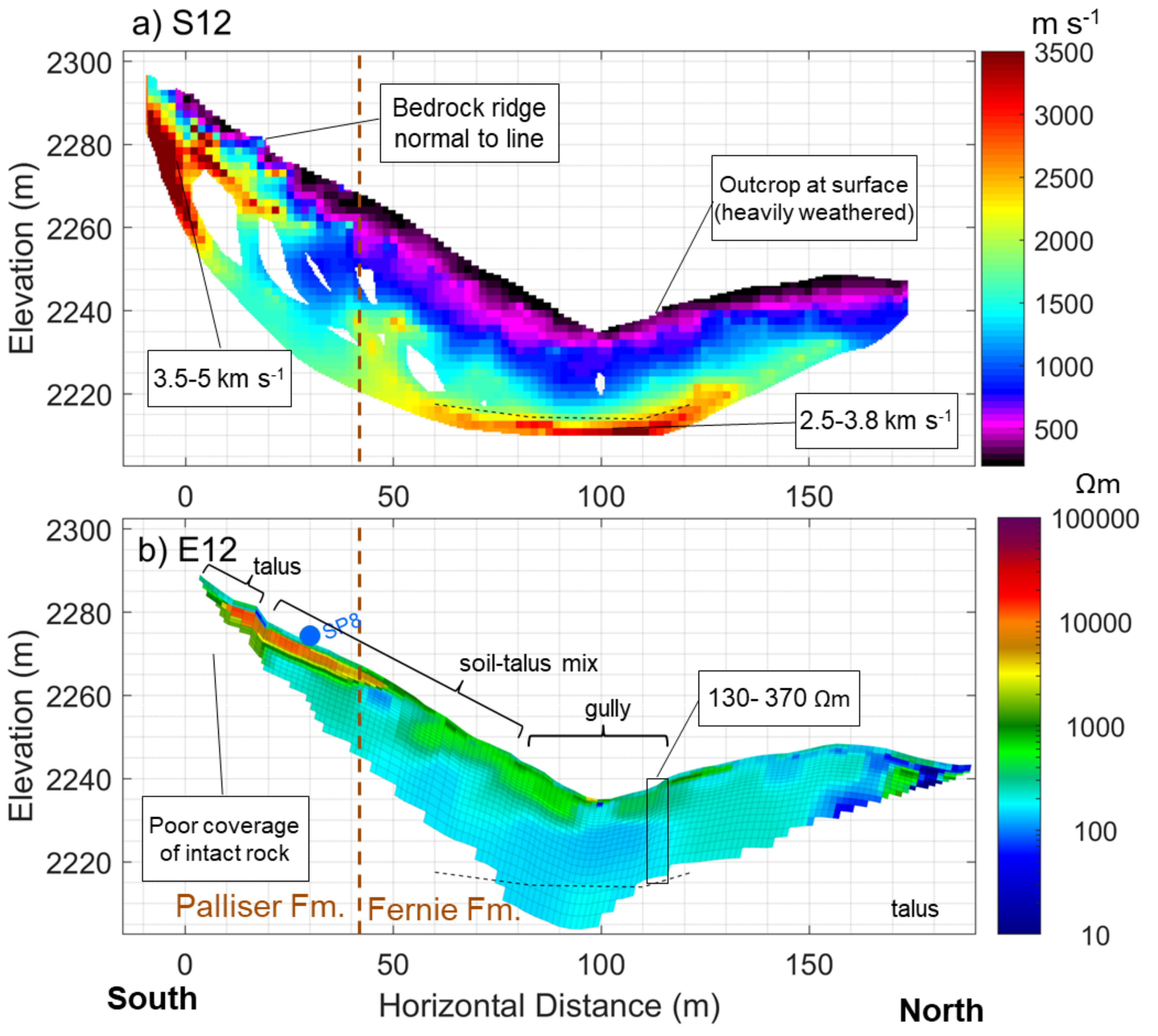

Figure S6 Composite of the (a) P-wave velocity model (RMS = 2.9 ms) and (b) resistivity model (absolute error = 9.7%) along Line 12 crossing two observed rock outcrops. Annotations include: descriptions of surface cover (black brackets), estimated location of the thrust fault from McMechan 2012 (dashed brown line at ~40 m), a spring (blue circle), and the estimated location of unweathered Fernie Formation (dashed black line).

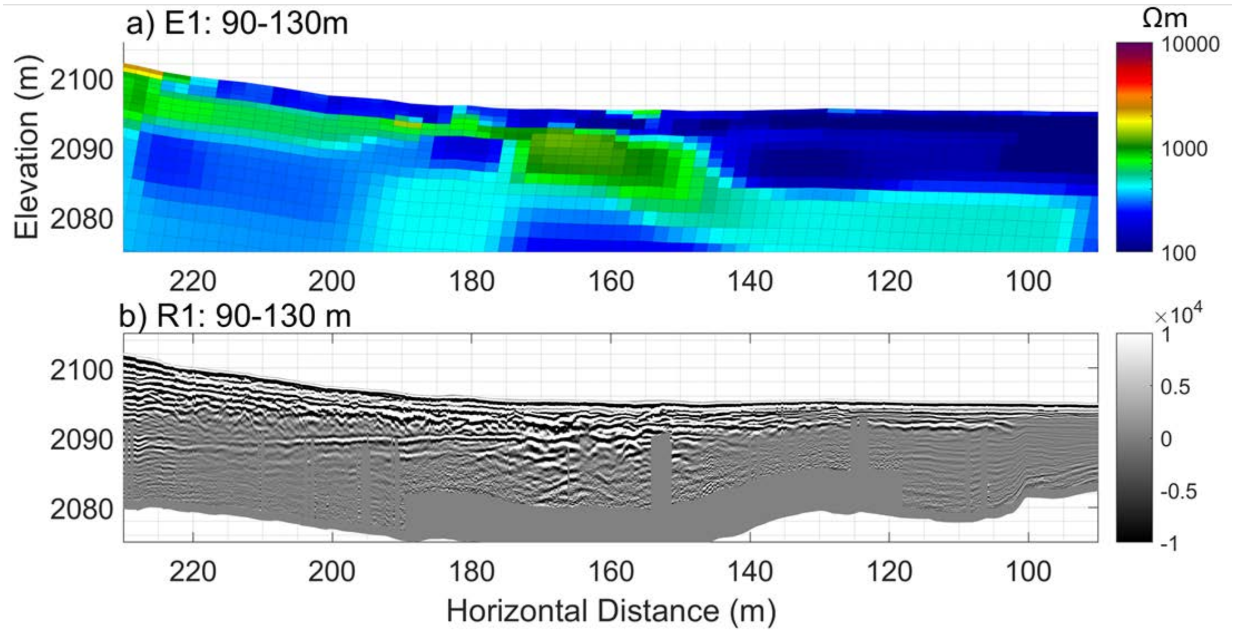

Figure S7: Zoomed view of the (a) resistivity and (b) radar images along Line 1. Note color scale limits in (a) above are narrower than those in Fig. 10b, making the resistivity contrasts between 180-230 m at 2090 masl more obvious.

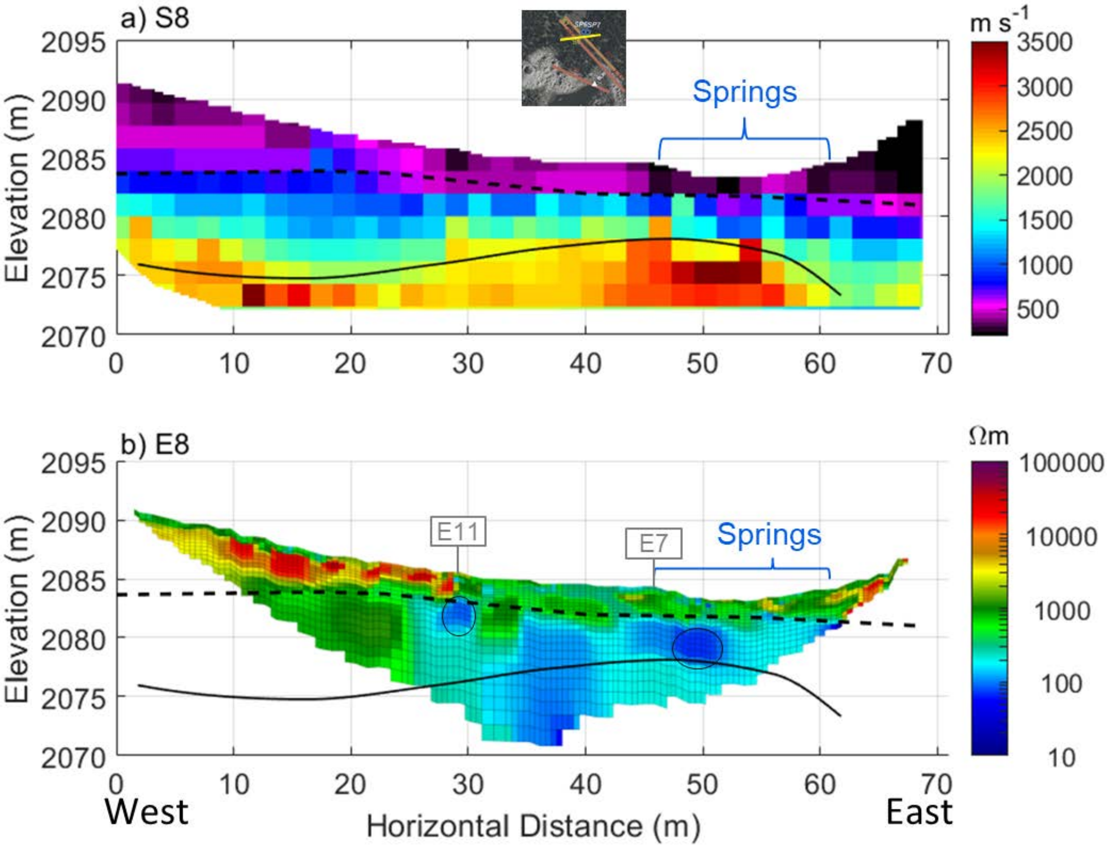

Figure S8: Geophysical models near the northern outlet spring: (a) along S8 (RMS = 4.6 ms) and (b) E8 (Absolute error = 13%). Annotations include: elevation of the interpreted depth to bedrock (solid black line), the interpreted depth to saturation (WT, dashed line), the location of the outlet springs SP6 and SP7, the intersection with ERT7 and ERT11 (grey boxes), and low-resistivity anomalies at depth (black ellipses).

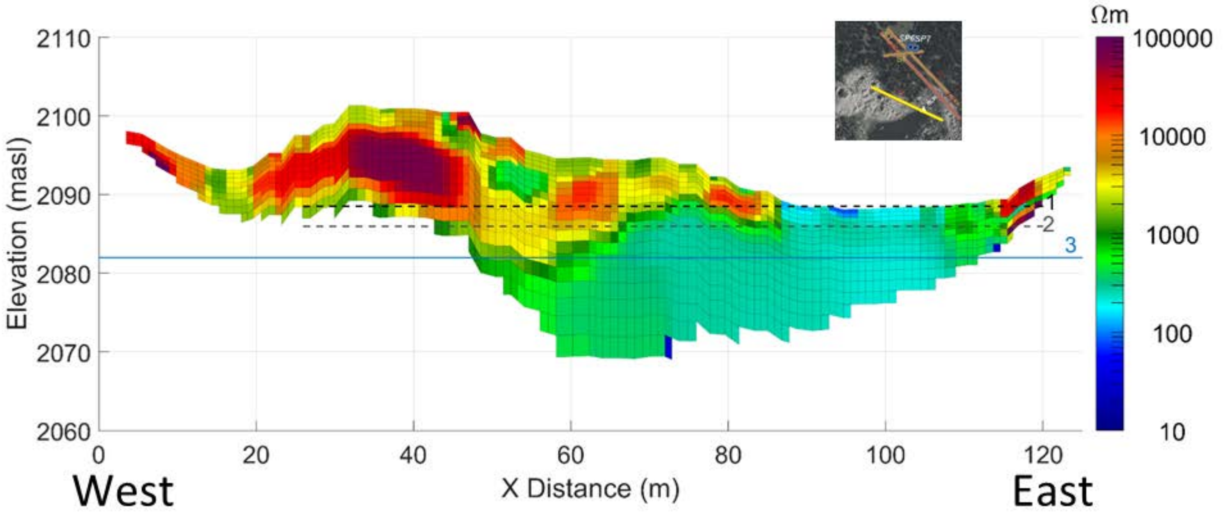

Figure S9: Resistivity image along Line 9 with absolute error of 15%. The annotated elevations are (1) The elevation of the lake at the time of survey (2088.5 masl), (2) the lowest point in the lake (2086 masl), and (3) outlet springs SP6 and SP7 (2082 masl).

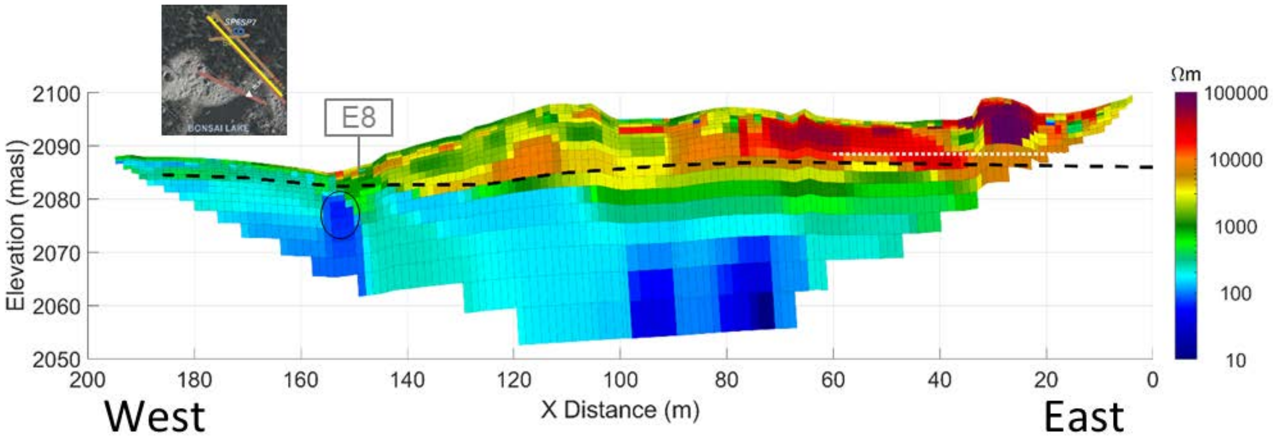

Figure S10: Electrical resistivity model along Line 11 with absolute error of 4.3%. Annotations note the elevation of the interpreted depth to saturation (black dashed line), the intersection with E8 (grey box), a low-resistivity anomaly at depth (black ellipse), and the elevation of Hathataga Lake (white dashed line 2088.5 masl) at the time of survey.

Supplementary Tables

Table S1 Summary of the attributes used by White (1981) to identify the dominant formation process for talus slopes.

	Slope angle	Grain attributes	Other attributes
**Rockfall talus**	• 35° to 45°	• Small rocks near the top<br>• Large ones with enough energy to flow down to toe of slope	• Angular rocks<br>• Usually lack vegetation
**Alluvial talus**	• 35° to 38° near the top<br>• ≤28° at the bottom and concave up	• Large rocks at top deposited where water loses energy with slope change<br>• Fines wash down in between coarser	• Fed by a couloir with sufficient source of water<br>• Vegetation<br>• Heavy storms or snowmelt may leave slush/debris flows, plus levees
**Avalanche talus**	• < 25°, concave up	• Any size, usually angular<br>• Often a fringe of coarser debris (Potter 1969)<br>• Rocks balanced in precarious positions at the bottom of the slope (Gardner 1970)	• Usually on lee-side slopes<br>• There can be scour strips

Table S2 Key survey parameters of all electrical resistivity tomography (ERT) lines collected.

Line name	Total length (m)	Nominal electrode spacing (m)	Orientation	Date measured	Number of electrodes
E1	572	4	E to W	2015-07-18	144
E2	188	4	S to N	2015-07-20	48
E3	424	8	W to E	2015-07-21	54
E4	236	4	S to N	2015-07-22	60
E5	284	4	N to S	2015-07-23	72
E6	142	2	E to W	2015-07-26	72
E7	142	2	E to W	2015-07-28	72
E8	177.5	2.5	W to E	2015-08-29	72
E9	71	1	W to E	2015-08-30	72
E11	213	3	E to W	2016-07-20	72

Table S3 Key parameters regarding the seismic data collected

Line name	Total length (m)	Nominal geophone spacing (m)	Orientation	Date acquired	Sampling interval (ms)	Acquisition time (s)
S1 East	286	2	E to W	2015-07-15, 2015-07-19	0.5 or 0.25	5 or 2
S1 West	96	2	E to W	2015-07-20	0.25	2
S2	96	2	S to N	2015-07-21	0.25	2
S3	224	2	W to E	2015-07-23, 2017-07-24	0.25	2
S4	190	2	S to N	2015-07-18	0.25	2
S5	142	2	N to S	2015-07-25	0.25	2
S6	142	2	E to W	2015-07-28	0.25	2
S7	177.5	2.5	W to E	2015-07-29	0.25	2
S8	71	1	N to S	2015-07-30	0.25	2

Appendix: Inversion Equations

The optimisation equation used in RES2DINV for inverting ERT data is (Loke et al. 2003):

$$\left(\mathbf{J}_i^{\mathrm{T}}\mathbf{R}_d\mathbf{J}_i + \lambda_i \boldsymbol{W}^{\mathrm{T}}\mathbf{R}_m\boldsymbol{W}\right)\Delta\mathbf{r}_i = \mathbf{J}_i^{\mathrm{T}}\mathbf{R}_d\mathbf{g}_i - \lambda_i \boldsymbol{W}^{\mathrm{T}}\mathbf{R}_m\boldsymbol{W}\mathbf{r}_{i-1} \qquad (1)$$

where

i	iteration number,
$\Delta\mathbf{r}_i$	change in model parameters (resistivity values)
$\mathbf{r}_{i-1}$	model resistivity values from the previous iteration
$\mathbf{g}_i$	data misfit vector
$\mathbf{J}$	Jacobian matrix of partial derivatives
$\mathbf{W}$	roughness filter (in this case, a first-order finite difference operator based on deGroot- Hedlin and Constable (1990))
λ_i	dampening factor for weighing the relative importance of the smoothness constraint.

The matrices $\mathbf{R}_d$ and $\mathbf{R}_m$, which are unused in the L_2-norm case, are added to ensure the elements of $\mathbf{g}_i$ and $\mathbf{W}$ have roughly equal weights during the optimization (Loke et al. 2003).

RES2DINV uses the mean absolute difference in logarithm between observed and measured resistivity values as their error metric (M. Loke, personal communication):

$$\%AbsoluteError = \frac{100\%}{N}\sum_{i=1}^{N}\left|\ln\left(\frac{\rho_{obs_i}}{\rho_{calc_i}}\right)\right| \qquad \mathbf{(2)}$$

where

N	number of measurements
ln	natural logarithm (logarithm of base e)
ρ_{obs}	observed (measured) apparent resistivity
ρ_{calc}	calculated apparent resistivity calculated based on the resistivity model

To invert the seismic refraction data, we use a code developed by Lanz et al. (1998). In general terms, the travel time of a seismic wave along a ray path S in a 2D isotropic medium is:

$$t = \int_S u(\boldsymbol{r}(\boldsymbol{x},\boldsymbol{z}))dr \qquad (3)$$

where

$\mathbf{r}(\mathbf{x}, \mathbf{z})$ position vector

$\mathrm{u}(\mathbf{r})$ the slowness field

The slowness field is approximated using a discrete, square grid with *m* cells, each with a constant slowness $\hat{u}_k$ $(k = 1 \ldots m)$. Hence, the ith travel time of *n* observations is:

$$t_i = \sum_{k=1}^{m} l_{ik}\hat{u}_k = \boldsymbol{L_i}\boldsymbol{\hat{u}} \quad (4)$$

where

l_{ik} portion of the ith raypath in the kth cell of u

The optimization equation is thus:

$$\begin{pmatrix} \boldsymbol{t} \\ \boldsymbol{h} \end{pmatrix} = \begin{pmatrix} \boldsymbol{L} \\ \boldsymbol{D} \end{pmatrix} \boldsymbol{\hat{u}} \quad (5)$$

where $\mathbf{h}$ and $\mathbf{D}$ represent the regularization parameters of the problem. These are used to (1) minimize the differences between the output slowness model and an input reference model $\boldsymbol{\hat{u}_0}$, and (2) impose smoothness constraints, wherein high spatial gradients in model slowness only appear where the data provide strong support for them. Mathematically, these are formulated as:

$$\begin{pmatrix} \lambda\beta\boldsymbol{\hat{u}_0} \\ \mathbf{0} \end{pmatrix} = \begin{pmatrix} \lambda\beta\boldsymbol{I} \\ \lambda(1-\beta)\boldsymbol{S} \end{pmatrix} \boldsymbol{\hat{u}} \quad (6)$$

where

I the identity matrix

S a Laplacian smoothing matrix from Ammon and Vidale (1993)

λ parameter controlling the overall amount of regularization applied

β parameter controlling the relative amount reference model dampening to smoothing, with a range of $0 < \beta < 1$

References

Ammon CJ, Vidale JE (1993) Tomography without rays. Bulletin of the Seismological Society of America 83:509-528.

deGroot-Hedlin C, Constable S (1990) Occam's inversion to generate smooth, two-dimensional models from magnetotelluric data. Geophysics 55:1613-1624. doi: 10.1190/1.1442813.

Gardner J. 1970. Geomorphic Significance of Avalanches in the Lake Louise Area, Alberta, Canada. Arctic and Alpine Research 2:135. DOI: 10.2307/1550348.

Loke MH, Acworth I, Dahlin T (2003) A comparison of smooth and blocky inversion methods in 2D electrical imaging surveys. Exploration Geophysics 34:182. doi: 10.1071/EG03182.

Potter N. 1969 Jan 1. Tree-Ring Dating of Snow Avalanche Tracks and the Geomorphic Activity of Avalanches, Northern Absaroka Mountains, Wyoming. Geological Society of America Special Papers 123:141–166. DOI: 10.1130/SPE123-p141.
